# Enzymatic depolymerization of alginate by two novel thermostable alginate lyases from *Rhodothermus marinus*

**DOI:** 10.3389/fpls.2022.981602

**Published:** 2022-09-20

**Authors:** Justyna M. Dobruchowska, Bryndis Bjornsdottir, Olafur H. Fridjonsson, Josef Altenbuchner, Hildegard Watzlawick, Gerrit J. Gerwig, Lubbert Dijkhuizen, Johannis P. Kamerling, Gudmundur O. Hreggvidsson

**Affiliations:** ^1^Microbial Physiology, Groningen Biomolecular Sciences and Biotechnology Institute (GBB), University of Groningen, Groningen, Netherlands; ^2^Matís Ltd., Reykjavík, Iceland; ^3^Institute for Industrial Genetics, University of Stuttgart, Stuttgart, Germany; ^4^Faculty of Life and Environmental Sciences, University of Iceland, Reykjavík, Iceland

**Keywords:** alginate oligosaccharides, thermophilic alginate lyase, guluronic acid, mannuronic acid, *Rhodothermus marinus*, NMR spectroscopy

## Abstract

Alginate (alginic acid) is a linear polysaccharide, wherein (1→4)-linked β-D-mannuronic acid and its C5 epimer, α-L-guluronic acid, are arranged in varying sequences. Alginate lyases catalyze the depolymerization of alginate, thereby cleaving the (1→4) glycosidic linkages between the monomers by a β-elimination mechanism, to yield unsaturated 4-deoxy-L-*erythro*-hex-4-enopyranosyluronic acid (Δ) at the non-reducing end of resulting oligosaccharides (α-L-*erythro* configuration) or, depending on the enzyme, the unsaturated monosaccharide itself. In solution, the released free unsaturated monomer product is further hydrated in a spontaneous (keto-enol tautomerization) process to form two cyclic stereoisomers. In this study, two alginate lyase genes, designated *alyRm3* and *alyRm4*, from the marine thermophilic bacterium *Rhodothermus marinus* (strain MAT378), were cloned and expressed in *Escherichia coli*. The recombinant enzymes were characterized, and their substrate specificity and product structures determined. AlyRm3 (PL39) and AlyRm4 (PL17) are among the most thermophilic and thermostable alginate lyases described to date with temperature optimum of activity at ∼75 and 81°C, respectively. The pH optimum of activity of AlyRm3 is ∼5.5 and AlyRm4 at pH 6.5. Detailed NMR analysis of the incubation products demonstrated that AlyRm3 is an endolytic lyase, while AlyRm4 is an exolytic lyase, cleaving monomers from the non-reducing end of oligo/poly-alginates.

## Introduction

**FIGURE 1:**
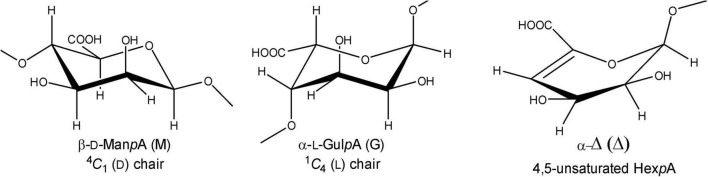
Structures of β-D-mannopyranosyluronic acid, α-L-gulopyranosyluronic acid, and 4-deoxy-α-L-*erythro*-hex-4-enopyranosyluronic acid units, as present in (enzymatically degraded) alginate.

Alginate (alginic acid) is the most abundant carbohydrate in several types of macroalgae, including marine brown seaweed (*Phaeophyceae*), and a major constituent of the algal cell wall and intracellular material (approximately 22–44% of the total cell dry weight) (Senturk Parreidt et al., 2018). It is a linear anionic polymer (molecular mass range 10–600 kDa) composed of (1→4)-linked β-D-mannopyranosyluronic acid (β-D-Man*p*A = M; ^4^*C*_1_ conformation) and (1→4)-linked α-L-gulopyranosyluronic acid (α-L-Gul*p*A = G; ^1^*C*_4_ conformation) units (Figure 1), arranged in varying sequences, i.e., block-regions of both homo- (M- or G-blocks) and hetero- (MG- or GM-blocks) polyuronic acid sequences (Penman and Sanderson, 1972; Gacesa, 1992; Pawar and Edgar, 2012). The constituting monomers β-D-Man*p*A and α-L-Gul*p*A are C-5 epimers. Alginate oligosaccharides, derived from brown seaweeds, are generally recognized as safe (GRAS) and non-toxic, and are widely used in the food, cosmetics, biochemical, and pharmaceutical industries due to their stabilizing, dehydrating, viscosifying, and gelling (with Ca^2+^) properties (Wong et al., 2000; Draget and Taylor, 2011; Zhang et al., 2021). Moreover, alginate oligosaccharides were demonstrated to have prebiotic activity (Wang et al., 2006). Alginate oligosaccharides and their derivatives have also been attracting attention due to their anti-inflammatory and anti-coagulant activities, and anti-tumor effects (Lee and Mooney, 2012; Chen et al., 2017; Xing et al., 2020). The constituting monomeric products of the lyase reactions may also be considered as important catalytic products as they can be converted into value-added chemicals, e.g., 2-keto-3-deoxy-gluconate (KDG). The application potential and the importance of the catalytic products of alginate is reviewed by Zhu and Yin (2015).

The enzymes, capable of catalyzing the depolymerization of alginic acid by splitting the glycosidic bonds through a β-elimination reaction, are generally known as alginate lyases (also called alginases, alginate depolymerases, transeliminases) (Gacesa, 1987; Wong et al., 2000; Kim et al., 2011; Ertesvåg, 2015). Three types of alginate-depolymerizing enzymes have been defined, i.e., poly(α-L-guluronate) lyase (EC 4.2.2.11), poly(β-D-mannuronate) lyase (EC 4.2.2.3), and bifunctional alginate lyase (Garron and Cygler, 2010; Zhu and Yin, 2015). Additionally, alginate lyases can have either endo- or exo-cleaving specificity. Cleavage of the **R**_**1**_-(1→4)-β-D-Man*p*A-**R**_**2**_ or **R**_**1**_-(1→4)-α-L-Gul*p*A-**R**_**2**_ glycosidic linkages (**R**_**1**_ and **R**_**2**_ = M- and/or G-containing saccharide) in the alginate polymers leads to a C-4,5 double bond in the D-mannuronate and/or the L-guluronate residue, yielding in both cases Δ-**R**_**2**_ with the new non-reducing terminal residue 4-deoxy-α-L-*erythro*-hex-4-enopyranosyluronic acid, designated by the symbol Δ (Figure 1; Gacesa, 1987, 1992).

A large number of alginate lyases, from sources ranging from various types of soil and marine bacteria, algae, and fungi to marine animals, have been characterized (Wong et al., 2000). In the Carbohydrate-Active Enzymes (CAZY) database (Drula et al., 2022),^1^ alginate lyases are assigned to different polysaccharide lyase (PL) families, based on amino acid sequence similarities. Several bacterial alginate lyase genes have been identified, cloned, and sequenced. Both native and recombinantly expressed enzymes have been characterized, giving insight into alginate lyase structure and function (Kim et al., 2009, 2011, 2012; Tøndervik et al., 2010; Hamza et al., 2011; Park et al., 2012). Few thermophilic alginate lyases have been described. A structure-function analysis of an alginate lyase from the marine thermophile *Defluviitalea phaphyphila* was recently reported. The enzyme, which was affiliated with a new PL family, PL39, had temperature optimum of activity at 65°C and pH optimum of activity at 5.8 (Wang et al., 2018; Ji et al., 2019). Another alginate lyase from *Nitratiruptor* sp. SB155-2, affiliated with family PL7, had a temperature optimum of activity at 70°C and pH optimum 6 (Inoue et al., 2016). Further, a recently described thermophilic exolytic alginate lyase of PL family 17, derived from an Arctic Mid-Ocean Ridge (AMOR) metagenomics data set, had pH optimum of activity at pH 6 and retained 100% activity after a 24-h incubation at 60°C (Arntzen et al., 2021).

Alginate lyases have been used for production of defined alginate oligomers and for protoplasting seaweed (Inoue et al., 2007; Chen et al., 2018). Their application for degradation of alginate polymers produced by *Pseudomonas aeruginosa* in cystic fibrosis patients has been studied (Mrsny et al., 1994; Alipour et al., 2009). Alginate lyases may also be used for the degradation of algal biomass in the production of biofuels or renewable commodity compounds (Jones and Mayfield, 2012; Wargacki et al., 2012; Doshi et al., 2016; Li et al., 2016, 2020). For utilization in those various industrial processes, robust alginate lyases which can function at extreme circumstances, such as elevated temperatures, may be of interest. Thermostability enables purification of recombinant enzymes from a mesophilic host by heat precipitation of the host’s proteins, which consequently lowers downstream production costs. Temperature is a major factor in defining the application range of an enzymatic activity, but optimum temperature of an industrial process is dependent on both substrate and products. High temperature (50–80°C) is optimal for polysaccharide degradation as it increases solubility, reduces viscosity of polysaccharides and facilitates enzymatic access. Thermostable enzymes have longer shelf lives and will tolerate prolonged reaction times and harsh conditions in bioconversion processes. Thermophilic enzymes may also streamline the integration of degradation processes with prior pre-treatment of seaweeds which is optimally carried out at elevated temperatures. Thermophilic and thermostable enzymes reduce the need for cooling from often high pre-processing temperatures of algal biomass that would be required for mesophilic enzymatic alginate degrading processes. High temperature also prevents contamination by spoilage bacteria (Turner et al., 2007).

The genome of the marine thermophile bacterium *Rhodothermus marinus* (strain MAT378) contains four alginate lyase genes, designated *alyRm1* to *alyRm4*. Two of the genes, encoding alginate lyases AlyRm1 and AlyRm2 belong to the CAZY polysaccharide lyase (PL) family PL6 subclass 2 and have been shown to function as endo-poly-MG lyases (Mathieu et al., 2016). This report describes the second two thermophilic alginate lyases, AlyRm3 and AlyRm4, their substrate specificity and products. AlyRm4 belongs to the PL17 and AlyRm3 belongs to a newly described polysaccharide lyase family, PL39. The encoding genes were identified in *Rhodothermus marinus*, a Gram-negative, aerobic, marine thermophile belonging to a sister phylum to *Bacteroidetes*, *Rhodothermaeota* (Munoz et al., 2016). The species which has been isolated from marine habitats around the world in the proximity of hot spring vents, is a known producer of various robust polysaccharide-degrading enzymes (Bjornsdottir et al., 2006; Ara et al., 2020). The thermophilic *R. marinus* alginate lyases with complementing activity add to the previously described activity range of alginate lyases and may have valuable industrial application properties.

The encoding genes *alyRm3* and *alyRm4* were amplified by PCR, cloned into expression vector and expressed in *E. coli*. The resulting recombinant enzymes, AlyRm3 and AlyRm4, were characterized with regard to thermostability, and temperature and pH optimum of activity and subsequently investigated in more detail for their depolymerizing activity on mannuronan (M-block alginate), guluronan (G-block alginate), and low-viscosity alginate from *Macrocystis pyrifera*. The complete reaction mixtures and the products, isolated by size-exclusion chromatography (SEC), were analyzed by TLC, MALDI-TOF-MS, GLC-EI-MS, and one-/two-dimensional ^1^H and ^13^C NMR spectroscopy.

## Materials and methods

### Cultivation, genome sequencing and analysis

*R. marinus* strain MAT378/R-21 is a close relative of the type strain *R. marinus* DSM4252. Both strains were originally isolated from a shallow submarine hot spring in SW Iceland (Alfredsson et al., 1988). The MAT378 strain was cultivated in RSC selective medium (Bjornsdottir et al., 2007), containing 0.2% soluble starch (Merck). Pure high-molecular-weight DNA and plasmid DNA was isolated using the MasterPure Gram Positive DNA Purification kit (Lucigen) according to manufacturer’s instructions and used for genome sequencing. The genome was originally sequenced by Sanger sequencing with approximately 5x coverage. With the emergence of high throughput sequencing, the genome was re-sequenced. Libraries were prepared using the Nextera Flex and Nextera MatePair methods (Illumina) and sequenced on the Illumina MiSeq platform. The generated sequence reads were assembled by using SPAdes assembler (Bankevich et al., 2012) and annotated by using the RAST annotation server (Overbeek et al., 2014).

### Identification of genes encoding alginate lyase

Putative alginate lyase encoding genes were identified by the NCBI BLAST program (non-redundant protein sequences database). Sequence Alignments were performed using the EBI ClustalW2—Multiple Sequence Alignment tool.^2^ Further protein domain prediction and classification into families was done using InterPro (EMBL-EBI) and SMART (EMBL) databases. Signal peptides were predicted using the SignalP-4.0 Server (CBS).

### Cloning, expression in *Escherichia coli* JM109 and purification

The putative *alginase lyase* genes, designated *alyRm3* and *alyRm4*, were amplified from the presented genome of strain MAT378 without the predicted signal peptide sequences (aa 1–17 and 1–22, respectively). For heterologous expression in *E. coli*, all the alginate lyases genes were modified with an N-terminal hexa-histidine tag. Primers were designed as shown in Supplementary Table 1 to amplify the coding regions of the respective genes and introducing the restriction sites *Bam*HI or *Bgl*II at the 5′ ends and a *Hin*dIII site behind the stop codons. The amplifications were performed using standard PCR conditions and a proofreading polymerase, the fragments cut with the corresponding restriction enzymes and inserted into the L-rhamnose inducible expression vector pJOE5751. The later contains a His6-eGFP fusion under control of the *rhaP*_*BAD*_ promoter. The single *Bam*HI and *Hin*dIII restriction sites in the vector allowed the replacement of the eGFP by the alginate lyase genes and fusion to the 5′ His6-tag. *E. coli* JM109 carrying the respective recombinant plasmids were cultivated in LB medium (200 mL), containing 100 μg/mL ampicillin. For expression of the genes, the cultures were grown at 37°C till cell density reached OD_600_ of 0.6 then induced by adding of 0.1% rhamnose and further grown for 4 h at 30°C. The cells were harvested by centrifugation at 4,500 × *g* for 20 min at 4°C, washed, resuspended in 10 mM potassium phosphate buffer, pH 6.5 and disrupted by passing them twice through a French press cell. After centrifugation (13,000 × *g* for 15 min at 4°C), the supernatants of the crude cell extracts and the cell pellets were analyzed by SDS-PAGE.

The purifications of recombinant alginate lyases proteins were performed by immobilized metal affinity chromatography (IMAC). The supernatant of the respective crude cell extract, containing approximately 25 mg *E. coli* protein, was applied onto 2 mL Talon^®^ metal affinity resin (Clontech) in a column using gravity flow. The resin was washed with 10 mL of washing buffer (50 mM potassium phosphate, 300 mM NaCl, 5 mM imidazole, pH 7.0). Bound protein was eluted with 3 mL of elution buffer (50 mM potassium phosphate, 300 mM NaCl, 150 mM imidazole, pH 7.0). (His)6-alginate lyase-containing fractions were combined and applied onto an NAP10 column (GE Healthcare), equilibrated with 50 mM potassium phosphate, 300 mM NaCl, pH 7.0 to remove imidazole and stored at 4°C.

### Protein determination and protein electrophoresis

Protein concentration was determined using Bradford reagent (Bio-Rad) and BSA standards for preparation of a standard curve. Sodium dodecyl sulfate polyacrylamide gel electrophoresis (SDS-PAGE) was performed using the method of Laemmli (1970), standard gels and a protein standard (Fermentas). Gels were stained using Coomassie Brilliant Blue R-250 (Sigma).

### Alginate substrate material

Sodium M- and G-block alginates with a broad range of degrees of polymerization (DP) [M-block (ALG500), averaged DP 20, M/G ratio > 4; G-block (ALG300), averaged DP 20, G/M ratio > 3] were purchased from Elicityl Oligotech*^R^* (Crolles, France). Low-viscosity sodium alginate from *Macrocystis pyrifera*, Kelp (Brookfield Viscosity 100–300 cps; 2% in H_2_O at 25°C) was purchased from Sigma-Aldrich (St. Louis, MO, United States).

### Measurements of lyase activity of AlyRm3 and AlyRm4

The temperature optimum and stability, pH optimum, and impact of NaCl concentration (salinity) were determined for each enzyme using the *M. pyrifera* alginate as substrate and applying the 3,5-dinitrosalicyclic acid (DNS) method for determination of reducing sugar (Miller, 1959). Generally, the reactions were run not more than 5 min of incubation time to minimize color formation from degradation products at temperatures above 50°C, which otherwise affected the results of the DNS assay. The reaction mixture consisted of 10 μL appropriate diluted purified enzyme, 80 μL 1.25% alginic acid and 10 μL 0.5 M potassium phosphate or acetate buffer (pH 5.5 and 6.5 for AlyRm3 and AlyRm4, respectively) with a final concentration of 50 mM. The temperature optimum experiments were performed at a range from 25 to 100°C. The temperature stability was determined by estimating the temperature at which 50% enzyme activity was measured after 30 min of incubation (T1/2 temperature). I.e., the pure enzymes (1 μM) were incubated in 0.1 M potassium buffer, pH 5.5 and 6.5 for AlyRm3 and AlyRm4, respectively, during 30 min at temperatures over a range from 40 to 95°C. Following incubation, the enzyme was cooled, and the remaining activity measured at 60°C, applying DNS assay. The effect of pH on enzyme activity was tested with purified enzymes using various buffers at 0.5 M: sodium acetate (pH 4.5–5.5), sodium/potassium phosphate (pH 6.0–8.0), and Tris-HCl (pH 8.5–9.5) under standard DNS assay conditions. The effect of NaCl on the alginate lyase activity of the purified enzymes was tested in 0.1 M potassium phosphate buffer at pH 7.0 for AlyRm4 and at pH 5.5 for AlyRm3, including different concentration of NaCl. All reactions were done in triplicates. The percent activity was determined from the absorption units subtracting the blank.

### Isolation and purification of product oligo-uronates

Preparative quantities of incubation mixtures [3 mL solutions, containing 300 μL (0.7 U/mL) enzyme (AlyRm3 or AlyRm4), 300 μL 0.5 M phosphate buffer, pH 5.5, and 2,400 μL alginate substrate (12.5 mg/mL; M-block, G-block, or *M. pyrifera* alginate), incubated for 24 h at 40°C] were fractionated by size-exclusion chromatography (SEC) on Bio-Gel P-2 (isolation of dimers), P-4 (isolation of trimers) or P-6 (isolation of tetramers) columns (90 × 1 cm; Bio-Rad, Richmond, CA). Elutions, with UV detection at 235 nm, were carried out with 0.2 M NH_4_HCO_3_ at room temperature and a flow rate of 12 mL/h, and 10 min-fractions were collected and lyophilized. Depending on the oligomer series (DP) according to MALDI-TOF-MS, some fractions were pooled for further separation on adequate Bio-Gel columns.

### Thin-layer chromatography

Carbohydrate samples were spotted in 1 cm lines on TLC sheets (Merck Kieselgel 60 F254, 20 × 20 cm), which were developed with 1-butanol: acetic acid: water = 2:1:1 (v/v/v). Bands were visualized by orcinol/sulfuric acid staining (10 min for 100°C).

### High performance anion-exchange chromatography

The distribution of the different oligomers in M-block and G-block (1.5 mg/mL, 25 μL) were analyzed on a Dionex DX500 workstation (Dionex, Amsterdam, Netherlands), equipped with an ED40 pulsed amperometric detection (PAD) system. The separation of oligosaccharides was carried out on a Ionpac AS4A column (250 × 5 mm; Dionex) by using a linear gradient of 25–300 mM sodium acetate in 100 mM NaOH (1 mL/min).

### Matrix-assisted laser-desorption ionization time-of-flight mass spectrometry

MALDI-TOF-MS experiments were performed using an Axima™ mass spectrometer (Shimadzu Kratos Inc., Manchester, United Kingdom), equipped with a nitrogen laser (337 nm, 3 ns pulse width). Negative-ion mode spectra were recorded using the reflector mode at a resolution of 5,000 Full Width at Half Maximum (FWHM) and delayed extraction (450 ns). The accelerating voltage was 19 kV with a grid voltage of 75.2%; the mirror voltage ratio was 1.12, and the acquisition mass range was 200–6,000 Da. Samples were prepared by mixing on the target 0.5 μL sample solutions with 0.5 μL aqueous 10% 2,5-dihydroxybenzoic acid as matrix solution.

### Gas-liquid chromatography—Electron impact mass spectrometry

Reaction mixtures, obtained after incubation of M-block alginate, G-block alginate, and *M. pyrifera* alginate substrates with AlyRm4 were lyophilized, and after trimethylsilylation [pyridine: hexamethyldisilazane: trimethylchlorosilane = 5:1:1 (v/v/v), 30 min at room temperature] analyzed by GLC-EI-MS. Screenings were carried out on a GCMS-QP2010 Plus instrument (Shimadzu Kratos Inc.) using an EC-1 column (30 m × 0.25 mm; Alltech/Grace, Deerfield, IL) and a temperature program of 140–250°C at 8°C/min (Kamerling and Gerwig, 2007).

### Nuclear magnetic resonance spectroscopy

Resolution-enhanced one-/two-dimensional (1D/2D) 500-MHz ^1^H/^13^C NMR spectra were recorded in D_2_O at pD 7.0 on a Varian Inova Spectrometer (NMR Center, University of Groningen) or on a Bruker DRX-500 Spectrometer (Bijvoet Center, Department of NMR Spectroscopy, Utrecht University) at probe temperatures of 300K or 334K. Before analysis, samples were exchanged twice in D_2_O (99.9 atom% D, Cambridge Isotope Laboratories, Inc., Andover, MA) with intermediate lyophilization, and then dissolved in 0.6 mL D_2_O. Suppression of the HOD signal using the Bruker instrument was achieved by applying a WEFT (water eliminated Fourier transform) pulse sequence for 1D experiments and by a pre-saturation of 1 s during the relaxation delay in 2D experiments. Standard “Presat” solvent signal suppression (WET1D pulse) was used in case of 1D experiments on the Varian instrument. The 2D gCOSY and TOCSY spectra were recorded using an MLEV-17 mixing sequence with spin-lock times of 20-200 ms. Natural abundance 2D ^13^C-^1^H HSQC experiments (^1^H frequency 500.0821 MHz, ^13^C frequency 125.7552 MHz) were recorded without decoupling during acquisition of the ^1^H Free Induction Decay (FID). All spectra were processed using MestReNova 5.3 (Mestrelabs Research SL, Santiago de Compostella, Spain). Chemical shifts (δ) are expressed in ppm by reference to internal acetone (δ 2.225 for ^1^H and δ 31.08 for ^13^C).

## Results

### Alginate utilization locus in *Rhodothermus marinus*

**FIGURE 2:**
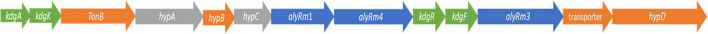
Alginate polysaccharide utilization locus (Alg-PUL) in *R. marinus* MAT378 containing the alginate lyase genes *alyRm1*, *alyRm3* and *alyRm4*. Annotation of the genes from left: *kdgA* (642 nt) encoding putative 4-hydroxy-2-oxoglutarate aldolase; *kdgK* (1,026 nt) encoding putative 2- dehydro-3-deoxygluconate kinase; *tonB* (2,805) TonB dependent receptor; *hypA* (2,181 nt) encoding T9SS type A sorting domain-containing protein; *hypB* (1,056 nt) encoding PorV/PorQ family protein; *hypC* (1,398 nt) encoding hypothetical protein; *alyRm1* (1,743 nt) encoding alginase; *alyRm4* (2,253 nt) encoding exo-alginase; *kdgR* (759 nt) encoding 4-hydroxy-2-oxoglutarate reductase; *kdgF* (357 nt) encoding cupin domain-containing protein. The *kdgR* (759 nt) and *kdgF* (366 nt) have been verified as genes encoding 4-deoxy-L-*erythro*-5-hexosulose uronate (DEHU) reductase and an enzyme catalyzing linearization of unsaturated uronates from alginate, respectively; *alyRm3* (2,673 nt) encoding endo-alginase; transporter gene (1,278 nt) encoding predicted mannuronate transporter; hypD (2,844 nt) encoding PD40 domain-containing protein. The colors of arrows indicate the functional role. Blue arrows; enzyme involved in degradation of alginate and production of uronic acids; Orange arrows: transport; Green arrows: enzymes for Entner Douderoff (ED) glycolytic processing of uronic acids. Gray arrows: hypothetical genes.

An alginate polysaccharide utilization locus (Alg-PUL) was identified following sequencing, assembly and annotation of the *Rhodothermus marinus* strain MAT378 genome as described in section “Materials and methods”. Structurally identical locus was identified in the reference strain, DSM4252, following publication of the genome sequence (Nolan et al., 2009). The locus contains three alginate lyase genes, designated *alyRm1* (PL6), *alyRm3* (PL39), and *alyRm4* (PL17) (Figure 2). An additional alginate lyase gene *alyRm2* (PL6) was identified outside the locus. The Alg-PUL also contained genes encoding catabolic enzymes, *kdgF* and *kdgR*, which activity has been verified (unpublished results), necessary for downstream funneling of final alginate degradation products into the Entner Douderoff pathway (unpublished data) as well as *kdgA* encoding putative 4-hydroxy-2-oxoglutarate aldolase; *kdgK* encoding putative 2-dehydro-3-deoxygluconate kinase. Relevant transporter genes and four hypothetical genes were also detected in the locus (Figure 2). The corresponding Alg-PUL genome sequence has been submitted to GeneBank with the accession number ON640824. The enzymes in *Rhodothermus marinus* DSM 4252 homologous to AlyRm4 and the AlyRm3 amino acid sequences have the GenBank numbers ACY48056.1 and ACY48059.1, respectively. Both have been annotated as Heparinase II/III family protein.

The predicted gene *alyRm3* ORF was 2,613 nt, encoding an 870 amino acid long polypeptide. Its calculated MW is 96,623 Da and pI 5.21. A signal sequence was predicted with cleavage site after Gln-17, a heparinase II/III-like protein domain (aa 374–518) and C-terminal associated with sorting to the outer membrane and covalent modification (Muth et al., 1998). A C-terminal sorting is also found in AlyRm1 PL6 alginate lyase encoded from same cluster indicating that both enzymes are extracellular. AlyRm3 can be assigned to a newly described polysaccharide lyase enzymes in the CAZY database, i.e., family PL39 (Ji et al., 2019). Closest relatives are found various lineages of the phylum of *Rhodothermaeota*, homologs having 50–100% aa identity depending on source species relatedness. AlyRm3 homologs are found in other *R. marinus* strains, e.g., the type strain DSM 4252. It is not found in *Rhodothermus bifrosti*, isolated from a terrestrial hot spring. The highest sequence similarity of AlyRm3, outside the genus was found with a heparinase II/III family protein from e.g., *Rhodocaloribacter litoris* (66% aa identity, GenBank: WP_166976759.1). Homologs are found in other phyla albeit with distinctly lower sequence similarity including a homolog from the clostridia species *Defluviitalea saccharophila* with 38% amino acid identity but with similar activity on alginate.

The predicted gene *alyRm4*, encoding AlyRm4, consists of 2,226 nt, which translates into a 742 aa polypeptide with MW of 83,633 Da and pI 6.06. A hydrophobic sequence was detected at the N-terminal, but the C- terminal sorting domain is missing indicating that the enzyme is located in the periplasmic space. Like AlyRm3, the enzyme contains a heparinase II/III-like protein domain (aa 386–539). AlyRm4 belongs to family PL17 of polysaccharide lyases and shows highest identity to homologous proteins in *Rhodothermaeota* and various species from other phyla including e.g., *Rhodocaloribacter litoris* WP166976762, 68% identity and *Vibrionales* bacterium SWAT-3 (45% aa identity, GenBank: ZP_01815528.1). Other thermophilic strains containing a PL17 sequence are *Rhodocaloribacter litoris, Spirochaeta thermophila*, *Merioribacter roseus*, and the *Paenibacillus* strain Y412MC10.

### Production and purification of the recombinant AlyRm3 and AlyRm4 enzymes

The encoding sequences of the mature enzymes were amplified by PCR and inserted into the L-rhamnose inducible expression vector pJOE5751 with a N-terminal fusion to a hexa-histidine sequence allowing production of His6-fusion proteins. Rhamnose induction of the *E. coli* JM109, harboring the respective plasmids, resulted in the production of high amounts of the recombinant proteins as identified by SDS-PAGE (Supplementary Figure 1). Compared to the uninduced crude extracts, a prominent protein band of the size of ∼95 kDa for AlyRm3 and ∼81 kDa for AlyRm4, respectively, indicated a tight regulation and high expression level. Recombinantly expressed alginate lyases were purified by IMAC to homogeneity as judged by SDS-PAGE (Supplementary Figure 1).

### Characterization of the recombinant alginate lyases

The recombinant alginate lyases AlyRm3 and AlyRm4 were active on alginic acid substrates but not on other substrates tested such as chondroitin and chitin (data not shown). The optimal pH, temperature optimum of activity and temperature stability are summarized in Table 1.

**TABLE 1:** Enzyme properties.

Alginate lyase	Temperature opt. (°C)	Temperature stability^a^ (°C)	pH opt.	NaCl opt.^b^(mM)
AlyRm3	75	T1/2 77°C – 40% remaining activity after 16 h at 70°C	5.5	0–800
AlyRm4	81	T1/2 78°C – 80% remaining activity after 16 h at 70°C	6.5	0–600

^a^T1/2 is the temperature at which 50% of optimum enzyme activity was measured after 30 min incubation. Also, the remaining activity (%) is shown following incubation for 16 h at 70°C. The activity was determined applying DNS assay.

^b^The salinity range where the enzymes show > 80% of optimum activity. The data is presented in Supplementary Figures 2, 3.

The enzymes are thermostable with temperature optima at 75° and 81°C for AlyRm3 and AlyRm4, respectively, measured at set conditions. The T_1/2_ (50% activity after 30 min incubation in buffer) was 77°C for AlyRm3 and 78°C for AlyRm4. The pH optimum of activity of AlyRm3 was 5.5. The pH activity range of AlyRm4 was 6.0–7.5 (>70%) with optimum at pH 6.5. As the thermophilic alginate lyases are derived from marine bacterium, the optimum salinity concentration was studied. Both enzymes were relatively stable at NaCl concentrations up to 1M (Supplementary Figures 2, 3).

### Substrate specificity of AlyRm3

In order to determine the substrate-depolymerizing specificity of the AlyRm3 enzyme, incubation experiments (0–24 h, pH 5.5, 40°C) were performed with M-block alginate, G-block alginate, and low-viscosity *M. pyrifera* alginate as substrates. Calculated by the peak integrals of specific protons in the ^1^H NMR spectrum, the low-viscosity *M. pyrifera* alginate contained an M:G ratio of 61:39. The enzymatic depolymerization reactions were followed by TLC, MALDI-TOF-MS, and ^1^H NMR spectroscopy.

**FIGURE 3:**
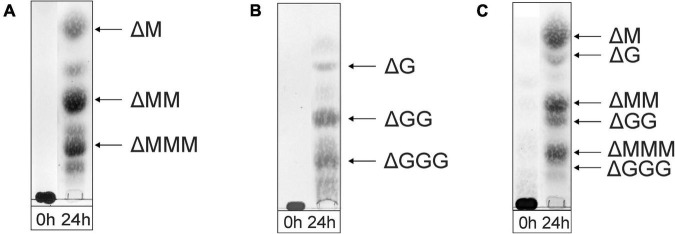
TLC analysis of the oligosaccharide mixtures generated after a 24-h incubation of **(A)** M-block alginate, **(B)** G-block alginate, and **(C)** low-viscosity *M. pyrifera* alginate with the AlyRm3 enzyme. Positions of isolated and identified products with non-reducing terminal 4-deoxy-α-L-*erythro*-hex-4-enopyranosyluronic acid (Δ) units are included (NMR analysis). Non-assigned bands are expected to stem from saturated oligosaccharides (MALDI-TOF-MS analysis). As the samples were run with several other samples, the 0-h control lanes and the lanes with the digested alginate samples were spliced for a clearer presentation.

As shown in the TLC analysis of the 24-h incubations (Figure 3), the three different substrates were depolymerized into mainly di-, tri-, and tetrasaccharides with a 4,5-unsaturated non-reducing-end uronic acid (Δ), indicating that the enzyme can cleave M-M, G-G, M-G, and G-M linkages *via* a β-elimination mechanism, which was confirmed by ^1^H NMR spectroscopy of isolated products (see below). Note that the starting materials have completely disappeared.

The negative-ion mode MALDI-TOF mass spectra of the three oligosaccharide mixtures (spectra not shown), obtained after a 24-h incubation, showed [M-H]^–^ peaks at *m/z* 351.0, 526.8, and 703.0, corresponding to di-, tri-, and tetrasaccharides, respectively, each with a 4,5-unsaturated non-reducing-end uronic acid (Δ). Furthermore, weak [M-H]^–^ peaks at *m/z* 369.2, 544.9, and 721.0 were also observed, in agreement with the presence of saturated di-, tri-, and tetrasaccharides, respectively. In the case of the G-block alginate incubation, additional small [M-H]^–^ peaks at *m/z* 879.0, 897.0, and 1073.3 were seen, corresponding with ΔGGGG, GGGGG, and GGGGGG, respectively.

To facilitate the NMR analysis of the oligosaccharide mixtures obtained after a 24-h incubation, generated unsaturated di-, tri-, and tetrasaccharides were isolated *via* fractionations on Bio-Gel P-2, P-4, and P-6, respectively. The 1D ^1^H NMR spectra of ΔM, ΔMM, ΔMMM, ΔG, and ΔGG are presented in Supplementary Figure 4. For detailed ^1^H and ^13^C assignments, gCOSY, TOCSY (mixing times 20, 50, 150, and 200 ms), and HSQC experiments were carried out. As an example, the 2D TOCSY (150 ms) and HSQC spectra of ΔM are presented in Supplementary Figure 5. The ^1^H/^13^C NMR chemical shift data of ΔG, ΔGG, ΔM, and ΔMM have been collected in Table 2. Note the specific Δ H-1 and Δ H-4 chemical shifts in case of Δ-M-**R** and Δ-G-**R** (**R** = M- and/or G-containing saccharide). Furthermore, it is of importance to note that the pH (pD) can influence chemical shifts, in particular the H-5 proton. Besides our own generated NMR chemical shift database, also previously published NMR data on alginate poly- and oligosaccharides were taken into account for resonance assignments (Grasdalen et al., 1979, 1981; Grasdalen, 1983; Steginsky et al., 1992; Heyraud et al., 1996; McIntyre et al., 1996; Zhang et al., 2004; Holtan et al., 2006; Li et al., 2011; Lundqvist et al., 2012; Hu et al., 2013).

**TABLE 2:** ^1^H and ^13^C chemical shifts^a^ of ΔM, ΔG, ΔMM, and ΔGG, recorded in D_2_O at 300K.

Saccharide	H-1/C-1	H-2/C-2	H-3/C-3	H-4/C-4	H-5/C-5
ΔMα	5.25/95.2	3.88/72.5	3.99/71.5	4.13/81.1	4.21/75.6
ΔMβ	4.92/95.6	3.97/73.5	3.78/74.0	3.80/80.9	3.98/78.3
Δ Mα	5.19/102.2	3.98/68.8	4.44/66.0	5.82/109.5	–/n.d.^b^
Δ Mβ	5.13/102.3	3.96/68.9	4.47/66.1	5.77/109.4	–/n.d.
ΔGα	5.23/96.4	3.90/68.1	n.d./n.d.	n.d./n.d.	n.d./n.d.
ΔGβ	4.89/95.5	3.58/71.2	4.20/72.7	4.17/82.4	4.42/75.6
Δ Gβ	5.22/102.5	3.92/68.7	4.33/64.9	5.92/109.8	–/n.d.
ΔMMα	5.25/95.3	3.92/72.3	3.96/71.5	4.02/80.3	4.14/75.1
ΔMMβ	4.93/95.7	3.97/72.3	3.77/73.7	3.75/80.1	3.95/78.3
ΔM Mα	4.70/103.0	4.01/72.6	3.76/73.7	3.91/80.2	3.75/78.1
ΔM Mβ	4.65/102.5	4.02/72.6	3.78/73.7	3.92/80.2	3.75/78.1
Δ MMα/β	5.13/102.1	3.93/67.7	4.46/64.9	5.76/109.7	–/n.d.
ΔGGα	5.23/95.8	n.d./n.d.	n.d./n.d.	n.d./n.d.	n.d./n.d.
ΔGGβ	4.88/95.5	3.63/71.1	4.15/72.1	4.03/82.4	4.42/75.6
ΔG Gα/β	5.04/102.9	3.87/66.8	4.10/71.0	4.25/81.9	4.49/69.4
Δ GGα/β	5.20/102.3	3.93/68.7	4.36/65.9	5.86/109.8	–/n.d.

The underlined residues correspond with the presented chemical shifts. For NMR spectra, see Supplementary Figures 4, 5.

^a^In ppm relative to internal acetone (δ 2.225 for ^1^H and δ 31.08 for ^13^C).

^b^n.d., not determined.

**FIGURE 4:**
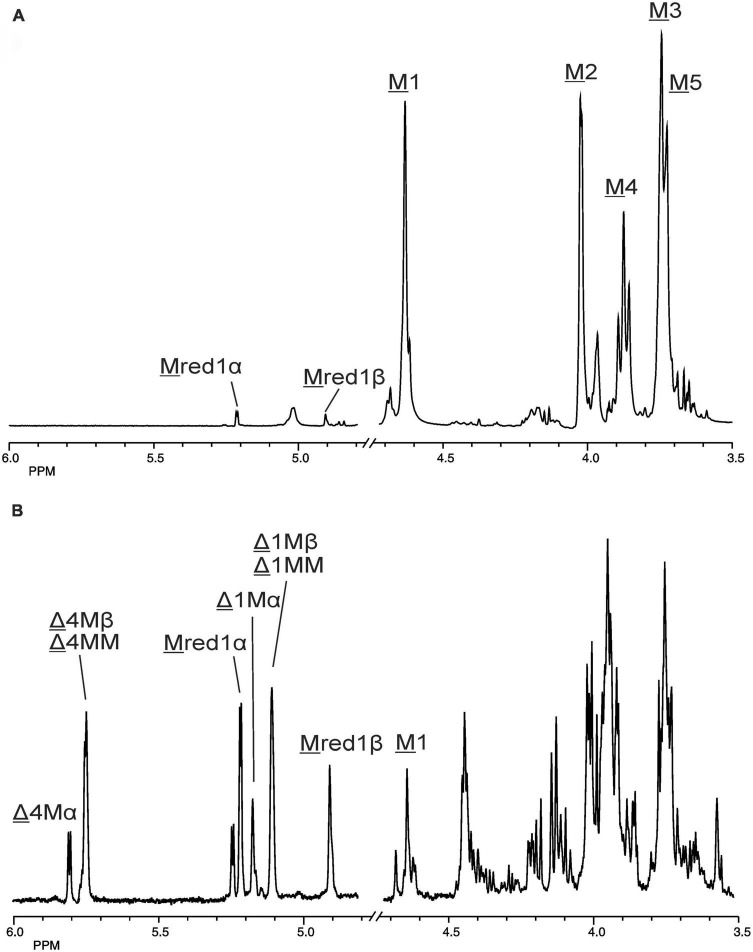
1D 500-MHz ^1^H NMR spectra (300K, D_2_O) of **(A)** the M-block alginate substrate before addition of the AlyRm3 enzyme, and **(B)** the oligosaccharide mixture generated after a 24-h incubation of the M-block alginate substrate with AlyRm3. M 1 to M 5 mean H-1 to H-5 of internal M residues; M red1α and M red1β mean H-1 of the reducing end α-M and β-M residue, respectively; Δ 4Mα/Δ 1Mα, Δ 4Mβ/Δ 1Mβ, and Δ 4MM/Δ 1MM mean H-4/H-1 of Δ in ΔMα, ΔMβ, and ΔM(M)n, respectively (for δ values, see Table 2).

**FIGURE 5:**
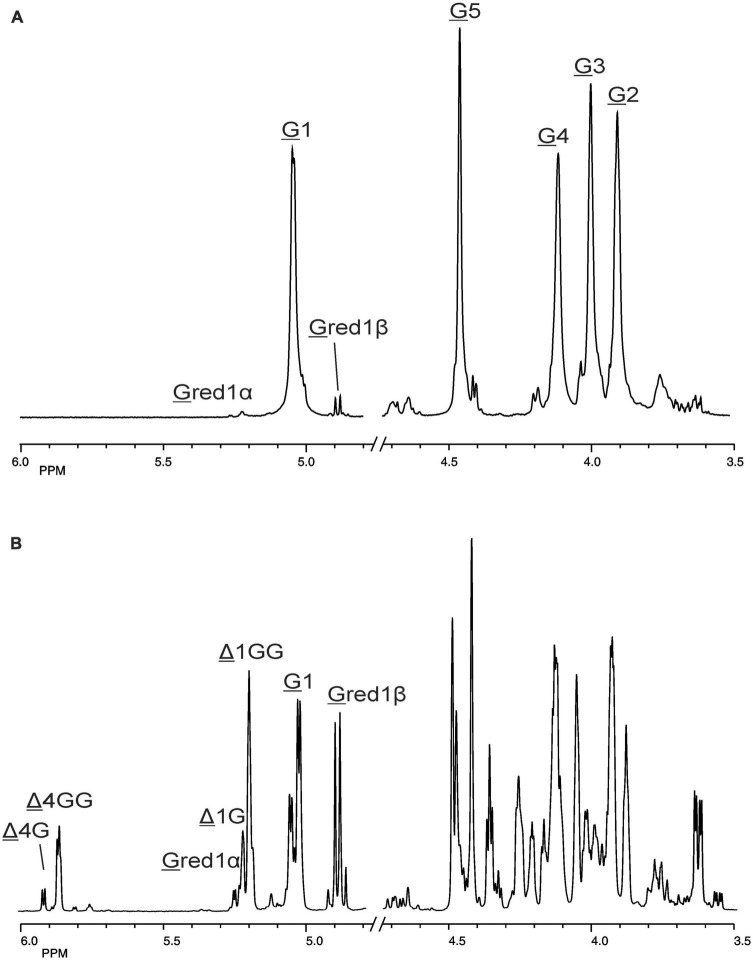
1D 500-MHz ^1^H NMR spectra (300K, D_2_O) of **(A)** the G-block alginate substrate before addition of the AlyRm3 enzyme, and **(B)** the oligosaccharide mixture generated after a 24-h incubation of the G-block alginate substrate with AlyRm3. Assignment according to Chen et al. (2018). G 1 to G 5 mean H-1 to H-5 of internal G residues; G red1α and G red1β mean H-1 of the reducing end α-G and β-G residue, respectively; Δ 4G/Δ 1G and Δ 4GG/Δ 1GG mean H-4/H-1 of Δ in ΔGβ and ΔG(G)_n_, respectively (for δ values, see Table 2).

The 500-MHz ^1^H NMR spectra at t = 0 h and t = 24 h of the M-block alginate incubation are presented in Figure 4, and those of the G-block alginate incubation in Figure 5. The average DPs (∼DPs) of the commercial M- and G-block alginates were checked by ^1^H NMR spectroscopy via integration (I) of the anomeric ^1^H signals in the respective spectra [∼DP_M–block_ = (I_M1_ + I_Mred1α+β_)/I_Mred1α+β_ and ∼DP_G–block_ = (I_G1_ + I_Gred1α+β_)/I_Gred1α+β_], yielding ∼DP_M–block_ 22 and ∼DP_G–block_ 25 (Figures 4A, 5A). The HPAEC-PAD profiles presented in Supplementary Figure 6, give an impression of the degree of polymerization or chain length distribution in M-block and G-block alginate. As is evident from the changes in the NMR profiles, going from Figure 4A to Figure 4B and from Figure 5A to Figure 5B, the enzyme is clearly acting as a lyase for both poly-M and poly-G homopolymeric regions. The formation of ΔM from the M-block alginate is demonstrated by the characteristic Δ H-4 resonances at δ 5.82 (coded Δ 4Mα) and δ 5.77 (coded Δ 4Mβ) (ratio ∼ 3:1), whereas the intense signal at δ 5.76 reflects the presence of ΔM(M)_n_ (coded Δ 4MM) (Figure 4B and Table 2; compare Supplementary Figure 4). Note that the MALDI-TOF-MS analysis showed mainly ΔM, ΔMM, and ΔMMM as end products (see above). For the G-block alginate, the appearance of Δ H-4 signals at δ 5.92 (coded Δ 4G) and δ 5.86 (coded Δ 4GG) indicated the presence of ΔG and ΔG(G)_n_, respectively (Figure 5B and Table 2; compare Supplementary Figure 4). Remarkably, the reducing end residue G (coded G red) has mainly β configuration, while the reducing end residue M (coded M red) showed a configuration ratio of α/β = 2:1. Noteworthy, MALDI-TOF-MS analysis showed mainly ΔG, ΔGG, and ΔGGG as end products.

**FIGURE 6:**
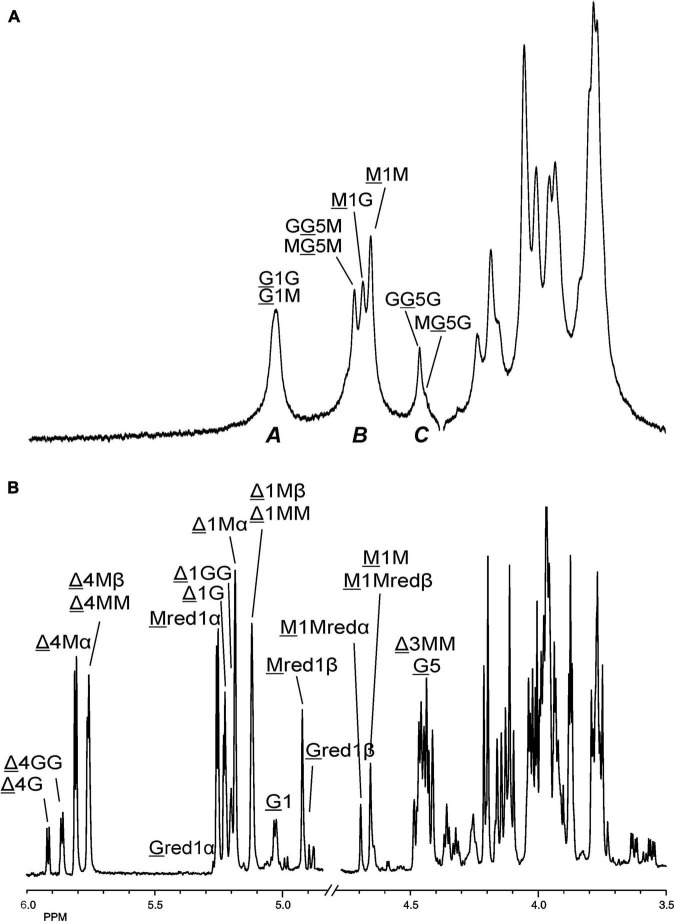
1D ^1^H NMR spectra of **(A)** (334K, D_2_O) M. pyrifera alginate substrate before addition of the AlyRm3 enzyme, and **(B)** (300K, D_2_O) the oligosaccharide mixture generated after a 24-h incubation of the alginate with the AlyRm3 enzyme. For the coding system in panel **(A)**, see text; for the coding system in panel **(B)**, see Figures 4, 5. M 1Mredα and M 1Mredβ/M 1M mean M H-1 of internal M-residues.

Making use of the NMR data obtained above for the M- and G-block alginate/oligosaccharide, the depolymerization of the low-viscosity alginate from M. pyrifera with the AlyRm3 enzyme was studied in more detail. The ^1^H NMR spectrum of the starting material, recorded at 334K instead of 300K to increase solubility and sharper resonance peak recovery (Figure 6A), showed in the δ 4.4-5.1 region the anomeric proton signals. The GulpA H-1 signals resonate at δ∼5.03 [(G or M)G 1G + (G or M)G 1M] and the ManpA H-1 signals at δ 4.70 (M 1G) and δ 4.66 (M 1M). The GulpA H-5 signals are present at δ 4.47 (GG 5G) with a shoulder at the right side for MG 5G (δ 4.43) and at δ 4.73 (MG 5M) with a shoulder at the left side for GG 5M (δ 4.76). Note that a preceding G residue (GG 5G vs. MG 5G and GG 5M vs. MG 5M) leads to a slightly higher chemical shift for G H-5, and that M H-1 in the M-G diad resonates at a slightly higher chemical shift than in the M-M diad (Grasdalen et al., 1979; Grasdalen, 1983). The ManpA H-5 signals resonate in the bulk region at δ∼3.75 (compare Figure 4). The M/G ratio was calculated from the relative integral areas (I) in the anomeric region, as described by Grasdalen et al. (1979), Grasdalen (1983) and Zhang et al. (2004). Hereto, the integration limits applied in the calculations of the area of the peak regions **A**, **B** and **C** were set to 4.97–5.10 ppm, 4.60–4.80 ppm, and 4.40–4.50 ppm, respectively (Figure 6A). Calculation of the percentage G and M monads in the M. pyrifera alginate, using the formula F_G_ = I**_A_** /I**_B_** + I**_C_**, resulted in 39% G and 61% M, and therefore a M/G ratio of 1.6. In a similar way, the percentage GG diads followed from the formula F_GG_ = I**_C_** /I**_B_** + I**_C_**, yielding 20% GG. As F_G_ = F_GG_ + F_GM_, F_M_ = F_MM_ + F_MG_, and F_GM_ = F_MG_, the other diads comprise 19% GM, 19% MG, and 42% MM.

The oligosaccharide mixture obtained after a 24-h incubation of the M. pyrifera alginate with the AlyRm3 enzyme was subjected to NMR analyses, and the assignments of resonance peaks were obtained by 2D TOCSY and HSQC experiments. The 1D ^1^H NMR spectrum (Figure 6B) showed anomeric signals for both reducing-end G and M residues. The G red1β resonance at δ 4.88 (J_1,2_ = 8.5 Hz) reflects a lyase activity of AlyRm3 on GM and/or GG diads, whereas the M red1α and M red1β resonances at δ 5.25 and δ 4.93, respectively, demonstrate such an activity on MM and/or MG diads (note that the M red1α signal at δ 5.25 overlapped the very minor G red1α resonance) (Table 2). Non-reducing-end Δ residues, formed via β-elimination from both M and G residues during the lyase cleavage reaction, were traced by their H-4 signals, thereby giving information about neighboring uronic acid residues. The Δ H-4 signals detected at δ 5.92 (Δ 4G) and δ 5.86 (Δ 4GG) indicate the presence of ΔG and ΔGG**R** -type oligosaccharides, respectively, whereas the Δ H-4 signals seen at δ 5.82/5.77 (Δ 4Mα/Δ 4Mβ) and δ 5.76 (Δ 4MM) revealed the presence of ΔM and ΔMM**R** -type oligosaccharides, respectively (**R** = M- and/or G-containing saccharide) (Table 2). The presence of Δ 4GM (δ 5.87) and Δ 4MG (δ 5.78) signals (Heyraud et al., 1996) could not be positively observed, due to partial signal overlap in the δ 5.75–5.95 region. Furthermore, the G H-5 signals (δ∼4.75) in the ^1^H NMR spectrum of the alginate substrate stemming from **R** MG 5M**R** and **R** GG 5M**R** parts (Figure 6A) have disappeared in the 24-h incubation mixture (Figure 6B). Therefore, the signal at δ 5.04 belongs to H-1 of internal G residues (G 1). The anomeric protons stemming from M residues next to the reducing end (e.g., ΔM M and ΔMM M) are observed at δ 4.70 (M 1Mredα) and δ 4.65 (M 1Mredβ), due to the α/β configuration of the reducing-end M residue. TLC (Figure 3) and MALDI-TOF-MS analyses supported the presence of ΔM, ΔMM, ΔMMM, ΔG, ΔGG, and ΔGGG in the product mixture after the 24-h incubation of M. pyrifera alginate with AlyRm3.

The Δ, G and M distribution (F) in the AlyRm3 lyase-degraded M. pyrifera alginate (original distribution: 39% G and 61% M; see above) was calculated by integration (I) of specific NMR peaks, using the following equations: F_G_=I_Gred__1α_+I_Gred__1β_+I_G__1_, F_M_=I_Mred__1α_+I_Mred__1β_+ I_M__1Mredα_+I_M__1Mredβ_ and F_Δ_=I_Δ__1G_+I_Δ__1GG_+I_Δ__1Mα_+I_Δ__1Mβ_+ I_Δ__1MM_ (or F_Δ_=I_Δ__4G_+I_Δ__4GG_+I_Δ__4Mα_+I_Δ__4Mβ+__Δ__4MM_). This resulted in 49% Δ, 9% G, and 42% M.

The larger decrease of G residues (39% → 9%) compared to the decrease of M residues (61% → 42%) might indicate a higher conversion of G units into non-reducing Δ residues. This could mean a preferential cleavage between **R** M-G**R** and/or **R** G-G**R** (**R** = M- and/or G-containing saccharide) by the AlyRm3 enzyme, despite the majority of M-M bonds in M. pyrifera alginate. The integrals of the M red1α/β signals (δ 5.25/4.93) predominated over those of the G red1α/β signals (δ 5.23/4.88), indicating a preferred splitting of the **R** M-GM**R** bonds over the **R** G-GM**R** bonds.

Moreover, the integrals ratio 4:1 of (Δ 4Mα + Δ 4Mβ + Δ 4MM) and (Δ 4G + Δ 4GG) supported the preferential cleavage between **R** M-GM**R** over **R** M-GG**R**, and eventually **R** G-GM**R** over **R** M-GG**R** (**R** = M- and/or G-containing saccharide). However, a higher rate of cleavage of **R** M-GM**R** bonds (yielding ΔG at the non-reducing ends and Mred at the reducing ends) is also possible, in view of the low presence of only 20% GG in M. pyrifera alginate (see above). Nevertheless, the results from the incubation of the M-block alginate (∼DP 22) with AlyRm3 (Figure 4) showed the enzyme’s capability of cleaving M-M bonds (yielding oligosaccharides having ΔM at the non-reducing ends and Mred at the reducing ends). The average degree of polymerization (DP_n_) of the products from the AlyRm3-degraded alginate substrate was estimated to be 2.3, using the following equation: DP_n_=(I_Δ1G_+I_Δ1M_+I_M1_+I_G1_+I_Mred_+I_Gred_)/[I_Δ 1G_+I_Δ 1M_+I_Mred_+I_Gred_)/2] (Ertesvåg et al., 1998). This is in reasonable agreement with the products, ranging from disaccharides to tetrasaccharides, as observed by TLC and MALDI-TOF-MS analysis.

The different observations indicated that within 24-h AlyRm3 can completely depolymerize M-block alginate (∼DP 22) into mainly ΔM, ΔMM, and ΔMMM (di-, tri-, and tetrasaccharides) and G-block alginate (∼DP 25) into ΔG, ΔGG, and ΔGGG (di-, tri-, and tetra-saccharides). So, for both M- and G-block alginates the high content of di-, tri-, and tetrasaccharides seems to indicate that the smallest substrate of the AlyRm3 is a tetrasaccharide, acting as a minimal recognition pattern. As deduced from the results of the incubations of AlyRm3 with M. pyrifera alginate, for alternating GMGM blocks, depolymerization mainly occurs into ΔG**R** due to the preferential cleaving of the **R** M-GM**R** bond (**R** = GMGMG-saccharide). So far, the following degradation pattern of alginic acid by AlyRm3 lyase can be proposed:

…M↓GMM↓GG↓GGG↓GGGG↓GM↓MMM↓MMMM↓ GM↓GM↓GMM↓M…

…M, ΔMM, ΔG, ΔGG, ΔGGG, ΔM, ΔMM, ΔMMM, ΔM, ΔM, ΔMM, Δ…

### Substrate specificity of AlyRm4

In a similar way, as described for the AlyRm3 enzyme, for the determination of the substrate specificity of the AlyRm4 enzyme, incubation experiments (0–24 h, pH 5.5, 40°C) were performed with M-block alginate (∼DP 22), G-block alginate (∼DP 25), and low-viscosity M. pyrifera alginate (M:G ratio of 61:39) as substrates. The enzymatic depolymerization reactions were followed by TLC and ^1^H NMR spectroscopy.

**FIGURE 7:**
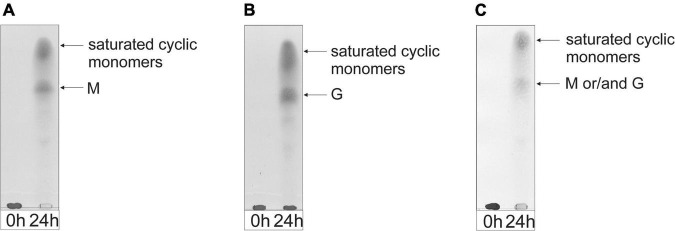
TLC analysis of the saccharide mixtures generated after a 24-h incubation of **(A)** M-block alginate, **(B)** G-block alginate, and **(C)** low-viscosity M. pyrifera alginate with the AlyRm4 enzyme.

TLC analysis of the 24-h incubations revealed that the enzyme can almost completely depolymerize the M-block and M. pyrifera alginates into monomers, as shown by the intense decrease of the spot at the origin on the TLC plate (Figures 7A,C). The G-block alginate, however, was only partially degraded into monomers after 24-h, as demonstrated by the left-over spot at the origin on the TLC plate (Figure 7B). Interestingly, no intermediate oligosaccharide bands could be detected, suggesting an exclusive exolytic activity of AlyRm4 on the three substrates. The assignment of the spots (Figure 7) correlated with isolated and identified products (see below).

The product mixtures, obtained after the 24-h incubations of the M-block, G-block, and M. pyrifera alginates, were further studied by ^1^H NMR spectroscopy (Supplementary Figure 7). In all three cases, the typical signals reflecting the presence of 4,5-unsaturated uronic acids (e.g., the Δ H-4 resonances in the δ 5.7–5.9 region) were absent, indicating, when compared with the AlyRm3 product mixtures (see Figures 4–6), a different depolymerization mechanism exists for AlyRm4.

**FIGURE 8:**
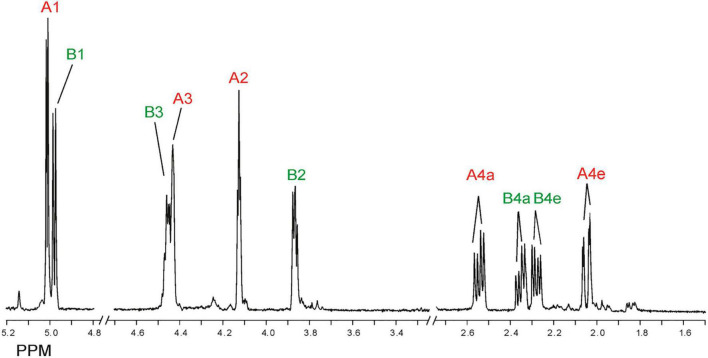
1D 500-MHz ^1^H NMR spectrum (300K, D_2_O) of Bio-Gel P-2 fraction II (cyclic stereoisomers A and B), obtained after 24-h of incubation of alginic acid with AlyRm4. Assigned according to Enquist-Newman et al. (2014). For 2D TOCSY and HSQC spectra, see Supplementary Figure 8. A1 means H-1 of structure A (red), etc., B1 means H-1 of structure B (green), etc. (for structures, see Figure 9).

**FIGURE 9:**
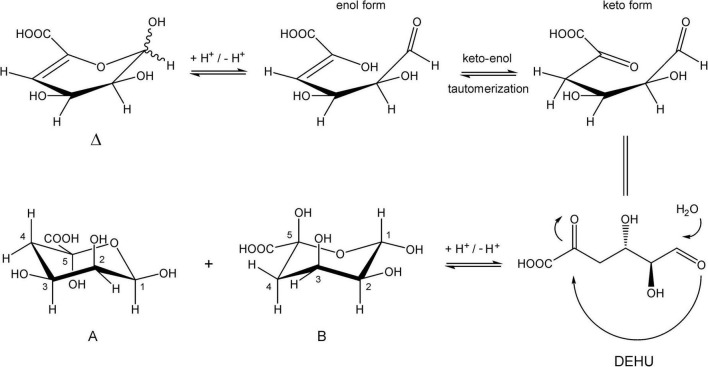
Conversion of 4-deoxy-L-erythro-hex-4-enopyranosyluronic acid into two cyclic hemiacetal structures in an aqueous solution.

Inspection of the 1D ^1^H NMR spectra of the product mixture of the three alginates (Supplementary Figure 7) showed only minor signals of residual free or reducing M (M red1α, δ 5.22; M red1β, δ 4.90) and/or free or reducing G (G red1α, δ 5.23; G red1β, δ 4.88). However, in the case of the G-block alginate incubation, intense signals (G1, δ 5.03; G2, δ 3.90; G3, δ 3.99; G4, δ 4.10; G5, δ 4.45) of remaining polymeric/oligomeric material were visible (Supplementary Figure 7B), supporting the TLC indications by the residual spot at the origin (Figure 7). To generate information about the origin of the intense signals, coded A and B in each NMR spectrum, the product mixture of the M. pyrifera alginate incubation was fractionated on Bio-Gel P-2, yielding two fractions denoted I and II. Fraction I contained free ManpA (M) and free GulpA (G) (NMR analysis; spectrum not shown), which could be explained as derived from non-reducing ends of different DP chains of the alginate substrate. Fraction II was studied by 1D/2D NMR spectroscopy (Figure 8 and Supplementary Figure 8) and GLC-EI-MS (Supplementary Figure 9), showing that the fraction contained two saturated cyclic monomers identical with 4-deoxy-L-erythro-5-hexosulose uronic acid [2,4,5,6-tetrahydroxy-pentahydro-pyran-2-carboxylic acid/1,2,3,5-tetrahydro-4H-pyran-5-carboxylc acid (TPC)]. As depicted in Figure 9, it is suggested that free Δ (4-deoxy-L-erythro-hex-4-enopyranosyluronic acid), released from alginic acid via an exolytic AlyRm4-catalyzed β-elimination reaction, is converted into its open chain form, which is further hydrated in a spontaneous process in aqueous solution to form the cyclic hemiacetal stereoisomers (Enquist-Newman et al., 2014). Endolytic activity is excluded because oligomers with Δ in a non-reducing position were not detected, which means that AlyRm4 cleaves monomers one by one from the reducing end. In this context, it should be noted that many lyases catalyze the hydration of bound terminal Δ units, with subsequent rearrangements, resulting in glycosidic bond cleavage (Enquist-Newman et al., 2014; Hobbs et al., 2016). If the enzyme would have a reductase activity, then the released Δ is non-enzymatically converted into 4-deoxy-L-erythro-5-hexosulose uronic acid (DEHU), then reduced to 2-keto-3-deoxy-D-gluconate (KDG), and further metabolized through the Entner-Douderoff pathway (Preiss and Ashwell, 1962a,b). However, in our case, the hypothesis that AlyRm4 has reductase activity can be excluded. The proton at C4 is added in a later spontaneous process (keto-enol tautomerization) in solution.

Note that, in theory, any stereochemical combination at C1 and C5 can take place during the ring closure process, yielding four products. As is clear from the ^1^H NMR spectrum (Figure 8), only two sets of ring protons are seen in a peak ratio of 1.0:0.8, indicating the presence of two main cyclic structures **A** (H1: δ 5.01, J_1,2_ 3.5 Hz) and **B** (H1: δ 4.98, J_1,2_ 6.0 Hz) in equilibrium. The full ^1^H and ^13^C NMR data of both stereoisomers **A** and **B** are presented in Table 3, and the TOCSY (200 ms) and HSQC spectra in Supplementary Figure 8.

**TABLE 3:** ^1^H and ^13^C chemical shifts^a^ and coupling constants^b^ of saturated cyclic structures A and B (Figure 9), generated by the incubation of M-block, G-block, and M. pyrifera alginates with AlyRm4, recorded in D_2_O at 300K.

H/C atoms	Structure A	Structure B
H-1	5.01 (J_1,2_ 4.0)	4.98 (J_1,2_ 6.0)
C-1	89.8	90.5
H-2	4.13 (J_2,1_ 3.5; J_2,3_ 3.5)	3.87 (J_2,1_ 5.5; J_2,3_ 4.5)
C-2	89.1	88.6
H-3	4.43 (J_3,2_ 3.0; J_3,4a_ 5.5; J_3,4e_ 3.0)	4.45 (J_3,4a_ 7.0; J_3,4e_ 6.0; J_3,2_ 4.5)
C-3	72.9	72.1
H-4a^c^	2.53 (J_4a,4e_ 14.0; J_4a,3_ 7.0)	2.35 (J_4a,4e_ 14.0; J_4a,3_ 7.0)
H-4e^c^	2.04 (J_4e,4a_ 14.0; J_4e,3_ 3.0)	2.28 (J_4e,4a_ 13.5; J_4e,3_ 6.0)

^a^In ppm relative to internal acetone (δ 2.225 for ^1^H and δ 31.08 for ^13^C).

^b^In Hz.

^c^a, axial; e, equatorial.

GLC-EI-MS analysis of the incubation mixture of the M. pyrifera alginate (24 h, 40°C), after direct lyophilization and trimethylsilylation, showed a major GLC peak (R_t_ 10.2 min) with a shoulder of which the EI-MS fragmentation patterns were in agreement with the trimethylsilylated (TMS) proposed six-membered structures **A** and **B** (MW 554 Da) (Supplementary Figure 9). Both stereoisomers could not be separated on the GLC column under the conditions used. The presence of phosphate buffer was reflected by the presence of the dominant GLC peak of trimethylsilylated phosphate [characteristic ions at m/z 314 (M) and 299 (M minus CH_3_)]. Surprisingly, a series of additional small peaks at the beginning of the gas chromatogram gave mass spectra that could be correlated with small organic acids products, such as glyceric acid, malic acid, (hydroxylated) glutaric acids and others (Supplementary Table 2 and Supplementary Figure 10). When the incubation of the M. pyrifera alginate with the AlyRm4 enzyme was performed at 65°C instead of 40°C, the GLC-EI-MS analysis revealed an increase of organic acid formation. It is suggested that the intermediate open chain 4-deoxy-L-erythro-hexosulose uronic acid (DEHU) in Figure 9 is the precursor of these non-enzymatic generation of organic acid products. The formation of high amounts of dicarboxylic acids was promoted by increasing the incubation temperature (data not shown). However, it still remains unclear how these products are generated and further study is in progress.

Finally, the ^1^H NMR spectrum of the G-block alginate, after a 24-h incubation with AlyRm4 at pH 5.5 and 40°C, showed the uncomplete depolymerization of the G-block by a broad signal of anomeric protons of internal G residues (G1) at δ 5.03, together with minor signals at δ 5.23 (G red1α) and δ 4.87 (G red1β) of Gα/β of non-degraded G-block alginate (Supplementary Figure 7B). Nevertheless, an exolytic activity of AlyRm4 is demonstrated by the presence of the signals belonging to the cyclic, saturated stereoisomers **A** and **B**, stemming from released reducing ends initially present at different length chains of the substrate (∼DP25).

Taking together, the absence of Δ in all three incubations with AlyRm4 clearly demonstrates that the lyase activity occurs only from the reducing end. Even when the G-block alginate was not completely degraded by the AlyRm4 enzyme, unsaturated oligomers with a Δ unit at the non-reducing end (e.g., ΔGGG) were not detected. Evidently, the free unsaturated monomers, released via β-elimination, are immediately converted to the two cyclic saturated monomers (**A** and **B**, Figure 9) and, eventually, into small organic acids, depending on the incubation temperature. As observed so far, all substrates were degraded, suggesting that the AlyRm4 enzyme has a broad substrate tolerance and can cleave M-M, M-G, G-M and G-G bonds, with a higher specificity/depolymerizing rate for MM homopolymeric sequences than for GG homopolymeric sequences. From the obtained analysis data, it can be concluded that the AlyRm4 lyase completely depolymerizes alginic acid into unsaturated monomers, which are mostly converted into cyclic monomers **A** and **B** and small organic acids, under the used conditions at elevated temperature:


$$\begin{array}{l} \text{G}↓\text{M}↓\text{G}↓\text{M}↓\text{M}↓\text{G}↓\text{G}↓\text{G}↓\text{G}↓\text{G}↓\text{G}↓\text{G}↓\text{G}↓\text{G}↓\text{G}↓\text{M}↓\text{M}↓\text{M}↓\text{M}↓\text{M}↓\text{M}↓\text{M}↓\text{M}↓\text{G}↓\text{M}↓\text{G}↓\text{M}↓.\;.\;.\\ \;↓\\ \text{G}△△△△△△△△△△△△△△△△△△△△△△△△△△.\;.\;.\\ \;↓\\ \text{ cyclic monomers }A\text{ and }B. \end{array}$$

## Discussion

Based on sequence similarities, alginate lyases have been assigned to the polysaccharide lyase families PL5, PL6, PL7, PL14, PL15, PL17, and PL18^3^ (Garron and Cygler, 2010; Zhu and Yin, 2015; Mathieu et al., 2016; Drula et al., 2022). Most bacterial alginate lyases, according to their primary structure, belong to the polysaccharide lyase families PL5 and PL7, and their molecular masses range from 25 to > 60 kDa (Lombard et al., 2010). The enzymes are generally classified according to their dominant typical cleaving action as poly-M lyase [(1→4)-β-D-mannuronan lyase; EC 4.2.2.3; PL5 (PL15 and PL17)] and poly-G lyase [(1→4)-α-L-guluronan lyase; EC 4.2.2.11; PL7] (Wong et al., 2000; Yamasaki et al., 2005; Kim et al., 2011). Furthermore, their three-dimensional arrangements allow grouping them into three structural classes, displaying either an α/α6 helix-barrel fold (Yoon et al., 1999) or a β-sandwich (jelly-roll) fold (Osawa et al., 2005). Several alginate lyases have been cloned and characterized from various sources (Zhu and Yin, 2015). In case of the alginate degrading thermophilic bacterium *R. marinus* strain MAT378/R-21, the (recombinant) AlyRm4 enzyme belongs to the PL17 polysaccharide lyase family, whereas the (recombinant) AlyRm3 enzyme belongs to the recently defined family PL39 (Ji et al., 2019).

Compared to other reported thermophilic alginate lyases, the recombinant AlyRm3 and AlyRm4 are highly thermostable, with T1/2, in absence of substrate, (50% activity after 30 min incubation in buffer) of 77°C and 78°C for AlyRm3 and AlyRm4, respectively. This makes them among the most thermostable alginates lyases that have been described to date, along with AMOR_PL17A, derived from an Arctic Mid-Ocean Ridge (AMOR) metagenomics data set (Arntzen et al., 2021).

Temperature and pH optimum, stability and optimum salinity determinations were carried out applying DNS assays, measuring the release of reducing sugars quantified as glucose equivalents. At higher temperatures (above 55°C), a dark brown color was formed in the reaction solutions and formation of dicarboxylic acids was detected. The DNS assay was adopted accordingly by limiting the time of the enzyme reaction. However, due to the color formation at high temperatures, measuring the specific enzyme activities close to temperature optimum applying DNS assay was considered imprecise, and not reported here.

The thermophilic AlyRm3 and AlyRm4 alginate lyases differ in depolymerizing activity, but together they cover a wide activity range. Our results showed that the endolytic AlyRm3 enzyme can cleave any of the four types of bonds (M-M, G-G, M-G, and G-M), with a slight preference for M-M bonds, and completely degrade alginate into unsaturated di-, tri-, and tetrasaccharides. Interestingly, the exolytic AlyRm4 lyase appears to cleave any of the two reducing end types (G and M) from alginate chains, releasing the unsaturated monosaccharide 4-deoxy-L-*erythro*-5-hexosulose uronic acid (DEHU), Δ, which spontaneously converts into cyclic monomers. Increasing the incubation temperature resulted in the formation of carboxyl organic acid products (Aoyagi et al., 2008). It must be noted that poly-MG lyases, showing exolytic activity, are very rarely found in nature.

The enzymes, described in this report, can be used as biocatalyst for saccharification of alginate, since they can efficiently degrade polyM, polyG, polyMG blocks, alginate oligosaccharides, and alginate to produce specific molecules with defined properties optimally suited for a given application (“tailor-made alginate”). The enzymatic degradation of alginate can be either selective or complete depending on the choice of enzymes. For instance, the recombinant AlyRm3 converted alginate into unsaturated di-, tri- and tetrasaccharides, while AlyRm4 depolymerized ∼95% of alginate into monomers. High efficiency of the reactions could be explained by the fact that the incubations were performed at elevated temperatures, where the solubility of alginate increases and viscosity decreases, facilitating enzymatic access. Robust alginate lyases, active in such extreme circumstances, may be of great interest for biofuel and chemical industries.

## Conclusion

The two alginate lyases described in this report are highly thermostable. Detailed NMR analysis of the incubation products demonstrated that AlyRm3 is an endolytic lyase, while AlyRm4, as an exolytic lyase, cleaves monomers from the non-reducing end of oligo/poly-alginates. The two enzymes can be used separately for selective partial degradation of alginate, or in combination for complete degradation of alginate for production of monomer residues. Their application in industrial processes, where higher processing temperatures are required or preferred, is of substantial interest.

## Data availability statement

The data presented in the study are deposited in the GenBank repository, accession number ON640824.

## Author contributions

All authors listed have made a substantial, direct, and intellectual contribution to the work, and approved it for publication.

## Footnotes

1
http://www.cazy.org/

2
http://www.ebi.ac.uk/

3
http://www.cazy.org

## Abbreviations

1D and 2D: one- and two-dimensional
DP: degree of polymerization
G: α -L-guluronic acid
g-COSY: gradient correlation spectroscopy
GLC-EI-MS: gas-liquid chromatography—electron impact mass spectrometry
GRAS: generally recognized as safe
HPAEC-PAD: high pH anion-exchange chromatography with pulsed amperometric detection
HSQC: ^1^H detected heteronuclear single quantum coherence spectroscopy
M: β -D-mannuronic acid
MALDI-TOF-MS: matrix-assisted laser desorption time-of-flight mass spectrometry
MLEV: composite pulse devised by M. Levitt
NMR: nuclear magnetic resonance
SEC: size-exclusion chromatography
TLC: thin-layer chromatography
TOCSY: total correlation spectroscopy
WEFT: water eliminated Fourier transform.
