# Supplementary material for: Enzymatic depolymerization of alginate by two novel thermostable alginate lyases from Rhodothermus marinus
Source: Front Plant Sci. 2022 Sep 20;13:981602. doi: 10.3389/fpls.2022.981602 (PMC9530828; doi:10.3389/fpls.2022.981602)
Supplement: Supplementary file 1 [file Table_1.pdf]

Supplementary Table S1. Alginase expression plasmids, alginase genes and primers for PCR amplification

Plasmid	Gene	Oligonucleotides used *
pHWG987	*alyRm3*	S8149: 5´-AAAGGATCCCAGAACCCTTATGAGACTTACACG
		S8150: 5´-AAAAAGCTTCTAAAATGCCAGACCCCGGAC
pHWG991	*alyRm4*	S8259: 5´-AAAGGATCCCTGGAAGTGCTCGCGCAGCC
		S8227: 5´- AAAAAGCTTAACGGCGGGAATCACTGGCAAC

*Restriction sites for cloning are underlined
