# Supplementary material for: Enzymatic depolymerization of alginate by two novel thermostable alginate lyases from Rhodothermus marinus
Source: Front Plant Sci. 2022 Sep 20;13:981602. doi: 10.3389/fpls.2022.981602 (PMC9530828; doi:10.3389/fpls.2022.981602)
Supplement: Supplementary file 2 [file Table_2.pdf]

Supplementary Table S2. Organic acids observed in the product mixture of *M. pyrifera* alginate after incubation with AlyRm4.

**Carboxylic acid**	**Rt**[a]	**Structure**	**MW (TMS)**	**MS**[b]	**EIMS fragments (*m/z*)**
Butanedioic acid (Succinic acid)	<3	HOOC-CH_2-CH_2-COOH	262		247, 218, 203, 157, 172, 147, 133, 129,116, 75, 73
Pentanedioic acid (Glutaric acid)	<3	HOOC-CH_2-CH_2-CH_2-COOH	276		261, 233, 204,186, 158, 147, 133, 129,116, 101, 97, 85, 75, 73, 55
Dihydroxy propionic acid (Glyceric acid)	3.2	CH_2OH-CHOH-COOH	322	(a)	307, 292, 217, 205, 189, 175,147, 133, 117, 103, 73
Monohydroxy butanedioic acid (Malic acid) (monohydroxy Succinic acid)	4.8	HOOC-CH_2-CHOH-COOH	350	(b)	335, 319, 307, 265, 245, 233, 217, 189, 175,147, 133, 117, 101, 73
Monohydroxy pentanedioic acid (monohydroxy Glutaric acid)	5.8	HOOC-CH_2-CH_2-CHOH-COOH	364	(c)	349, 321, 274, 259, 247, 231, 203, 157, 147, 129, 116, 73
Dihydroxy pentanedioic acid (dihydroxy Glutaric acid)	7.5	HOOC-CHOH-CH_2-CHOH-COOH	452	(d)	437, 347, 335, 319, 305, 292, 257, 245, 233, 220, 189, 147, 73
Trihydroxy pentanedioic acid (trihydroxy Glutaric acid)	9.1	HOOC-CHOH-CHOH-CHOH-COOH	540		525, 435, 423, 407, 361, 333, 321, 292, 277, 221, 189, 147, 73
Trihydroxy hexanedioic acid	9.9	HOOC-CHOH-CHOH-CHOH-CH_2-COOH	554		539, 449, 421, 407, 347, 335, 305, 292, 245, 233, 217, 189, 147, 73

[a] Rt is GLC retention time (min) on EC-1 (140-250 ºC at 8 ºC/min). [b] (a) – (d) refer to the EI mass spectra in Supplementary Figure S10.
