# Supplementary material for: Enzymatic depolymerization of alginate by two novel thermostable alginate lyases from Rhodothermus marinus
Source: Front Plant Sci. 2022 Sep 20;13:981602. doi: 10.3389/fpls.2022.981602 (PMC9530828; doi:10.3389/fpls.2022.981602)
Supplement: Supplementary file 3 [file Image_1.pdf]

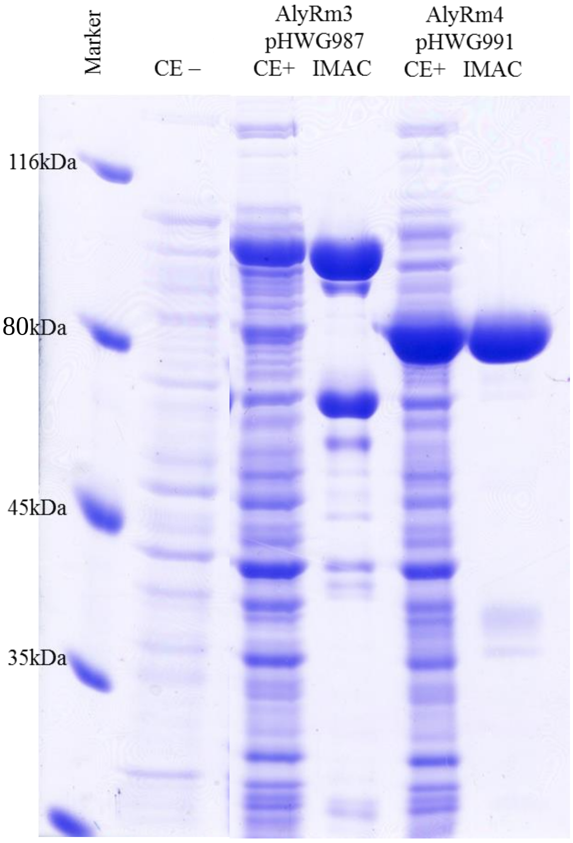

Supplementary Figure S1. 10% SDS-PAGE of crude extracts (15 µg protein) of *E. coli* JM109 harbouring the respective plasmids, pHWG987 and pHWG991 containing the alginase lyase genes *alyRM3* and *alyRM4*, respectively, and purified $(His)_6$-alginate lyases (3µg protein) after IMAC. (CE- crude extract without induction; CE+ crude cell extract from rhamnose-induced cells). The first two lanes showing the marker and the control crude extract were spliced to the lanes with the alginase crude extracts and the purified enzymes for a clearer presentation
