# Supplementary material for: Enzymatic depolymerization of alginate by two novel thermostable alginate lyases from Rhodothermus marinus
Source: Front Plant Sci. 2022 Sep 20;13:981602. doi: 10.3389/fpls.2022.981602 (PMC9530828; doi:10.3389/fpls.2022.981602)
Supplement: Supplementary file 4 [file Image_2.pdf]

(A)

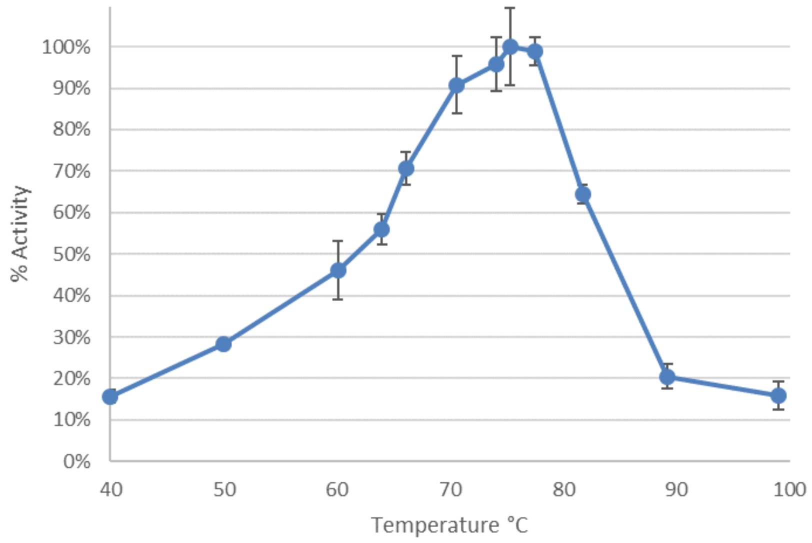
100%
90%
80%
70%
60%
50%
40%
30%
20%
10%
0%
% Activity
40
50
60
70
80
90
100
Temperature °C

(B)

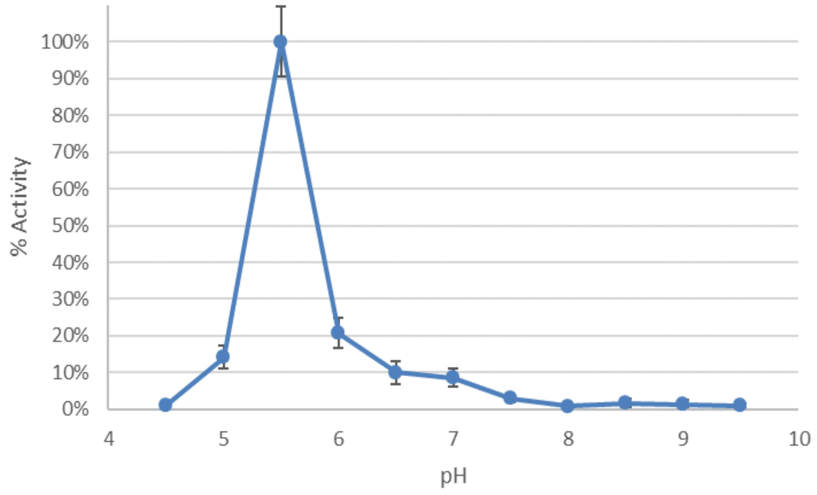
100%
90%
80%
70%
60%
50%
40%
30%
20%
10%
0%
% Activity
4
5
6
7
8
9
10
pH

(C)

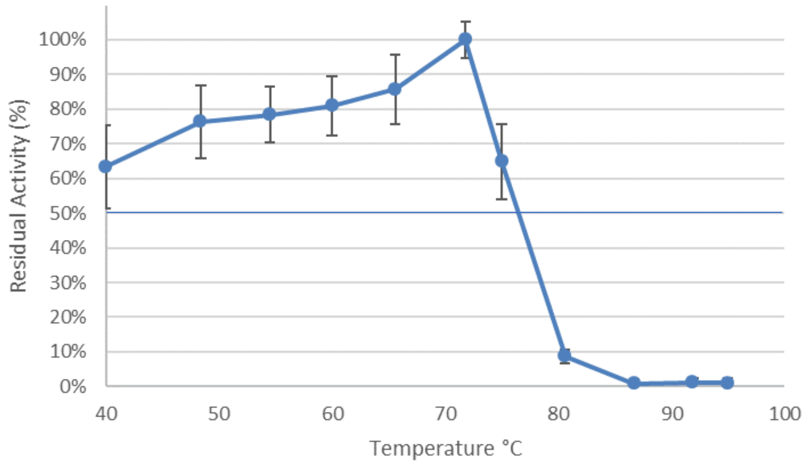
100%
90%
80%
70%
60%
50%
40%
30%
20%
10%
0%
Residual Activity (%)
40
50
60
70
80
90
100
Temperature °C

(D)

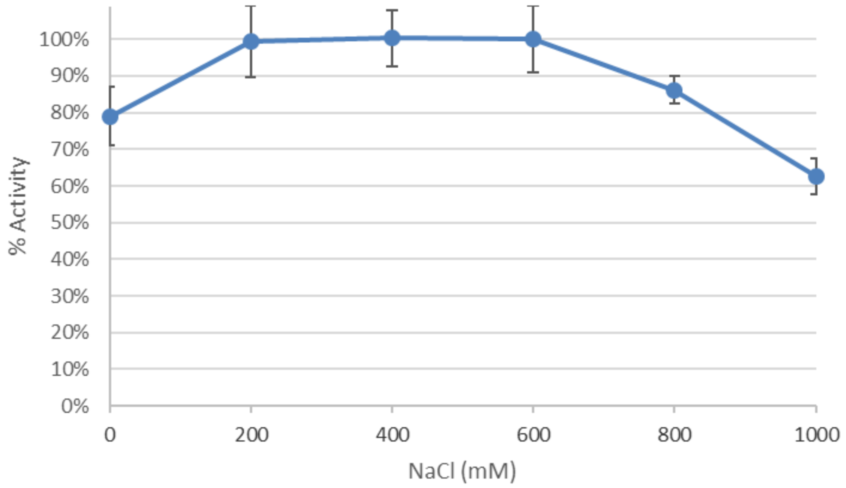

Supplementary Figure S2. Characterization of recombinantly produced AlyRm3 carried out applying DNS assay. The reaction time was 1 minute to minimize colour formation of mono-uronate derivatives. The reactions were done in triplicate. The average values (dots) and standard deviations (error bars) are shown. A) Optimum temperature of activity, B) Thermal stability in absence of substrate. T1/2 determined by incubating the enzyme (1 µM) in buffer, at temperatures between 40°C and 95°C for 30 minutes and subsequently measuring the residual activity at 60°C, C) pH activity profile, and D) Optimum salinity.
