# Supplementary material for: Enzymatic depolymerization of alginate by two novel thermostable alginate lyases from Rhodothermus marinus
Source: Front Plant Sci. 2022 Sep 20;13:981602. doi: 10.3389/fpls.2022.981602 (PMC9530828; doi:10.3389/fpls.2022.981602)
Supplement: Supplementary file 6 [file Image_4.pdf]

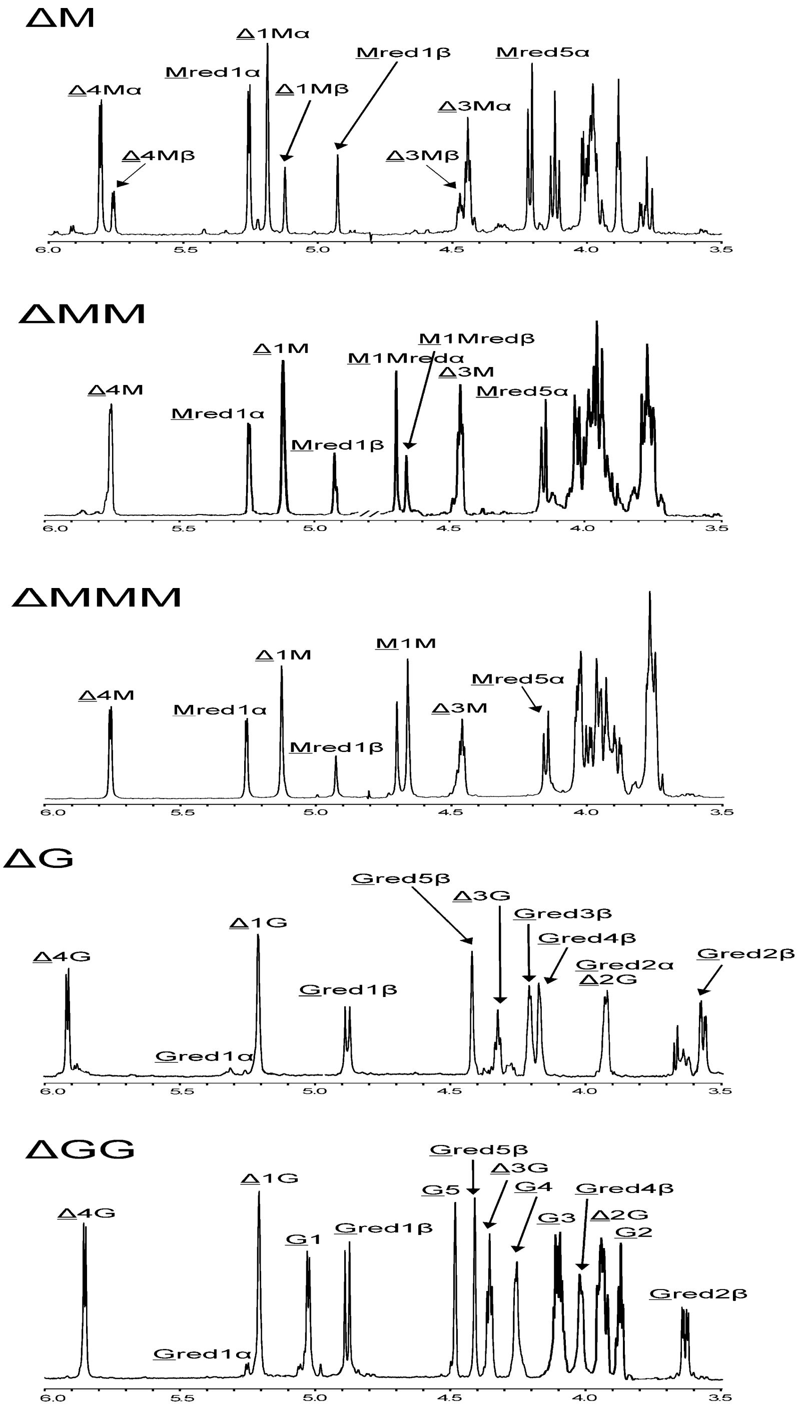

Supplementary Figure S4. 500-MHz ^{1}H NMR spectra of isolated ΔM, ΔMM, ΔMMM, ΔG, and ΔGG, recorded in D_2O at 300K (See also Supplementary Fig S5). Δ4Mα, Δ4Mβ, and Δ4M mean Δ H-4 of the presented saccharides, etc.; Mred1α and Mred1β mean M H-1 of the reducing-end α-M and β-M residue, respectively, in the presented saccharides, etc.; M1Mredα, M1Mredβ, and M1M mean M H-1 of internal M-residues in the presented tri or tetrasaccharide, etc.; Gred1α and Gred1β mean G H-1 of the reducing-end α-G and β-G residue, respectively, in the presented saccharides, etc.; G1 means G H-1 of the internal G-residue in the presented trisaccharide, etc. (see Table 2 in main text).
