# Supplementary material for: Enzymatic depolymerization of alginate by two novel thermostable alginate lyases from Rhodothermus marinus
Source: Front Plant Sci. 2022 Sep 20;13:981602. doi: 10.3389/fpls.2022.981602 (PMC9530828; doi:10.3389/fpls.2022.981602)
Supplement: Supplementary file 7 [file Image_5.pdf]

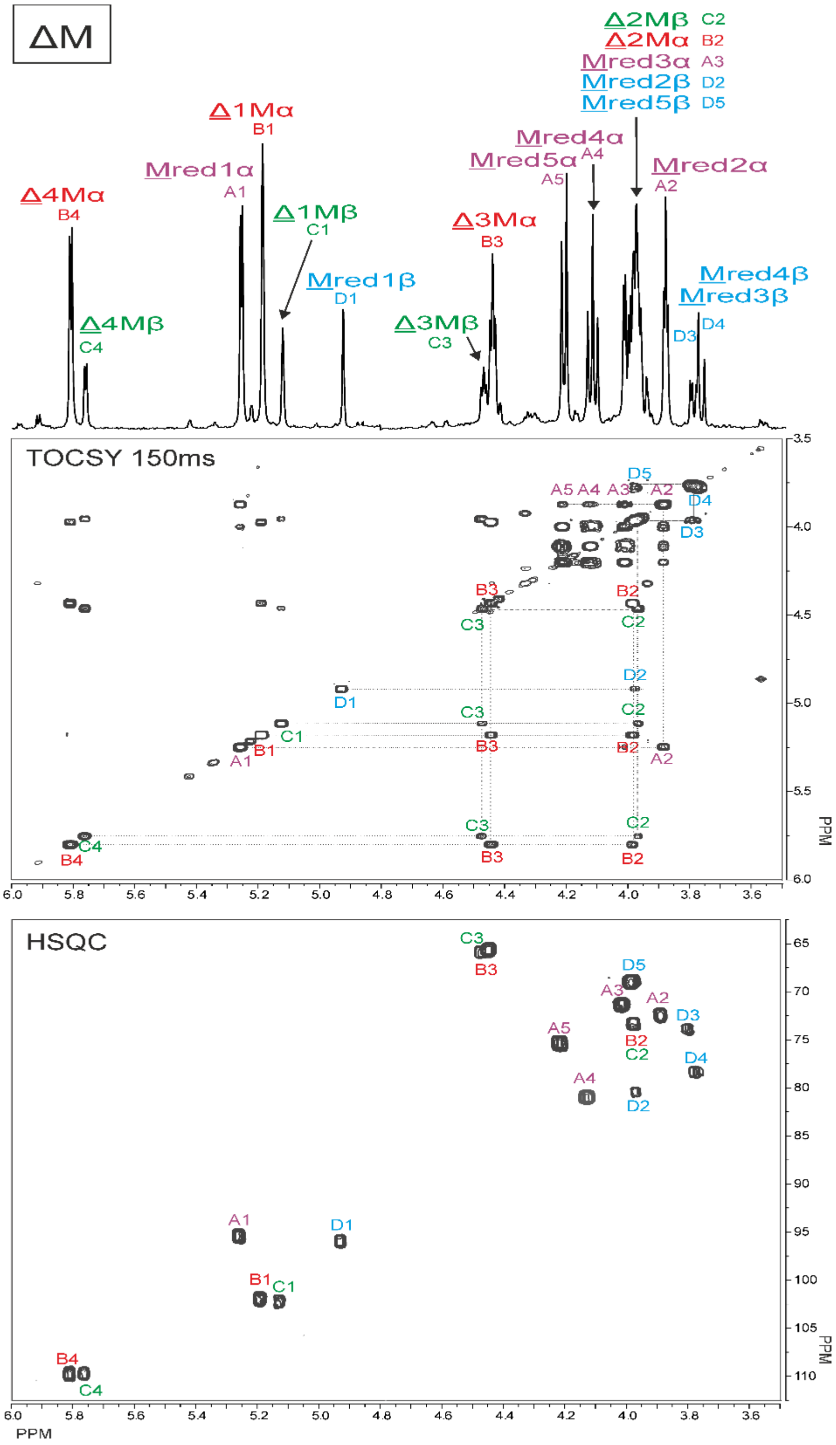

Supplementary Figure S5. ^{1}H, TOCSY (150 ms), and HSQC spectra of disaccharide ΔM (in fact, two disaccharides ΔMα and ΔMβ), recorded in D_2O at 300K. Δ4Mα means Δ H-4 of disaccharide ΔMα, Δ4Mβ means Δ H-4 of disaccharide ΔMβ, etc.; Mred1α means M H-1 of disaccharide ΔMα, Mred1β means M H-1 of disaccharide ΔMβ, etc. (see Table 2 in main text).
