# Supplementary material for: Enzymatic depolymerization of alginate by two novel thermostable alginate lyases from Rhodothermus marinus
Source: Front Plant Sci. 2022 Sep 20;13:981602. doi: 10.3389/fpls.2022.981602 (PMC9530828; doi:10.3389/fpls.2022.981602)
Supplement: Supplementary file 8 [file Image_6.pdf]

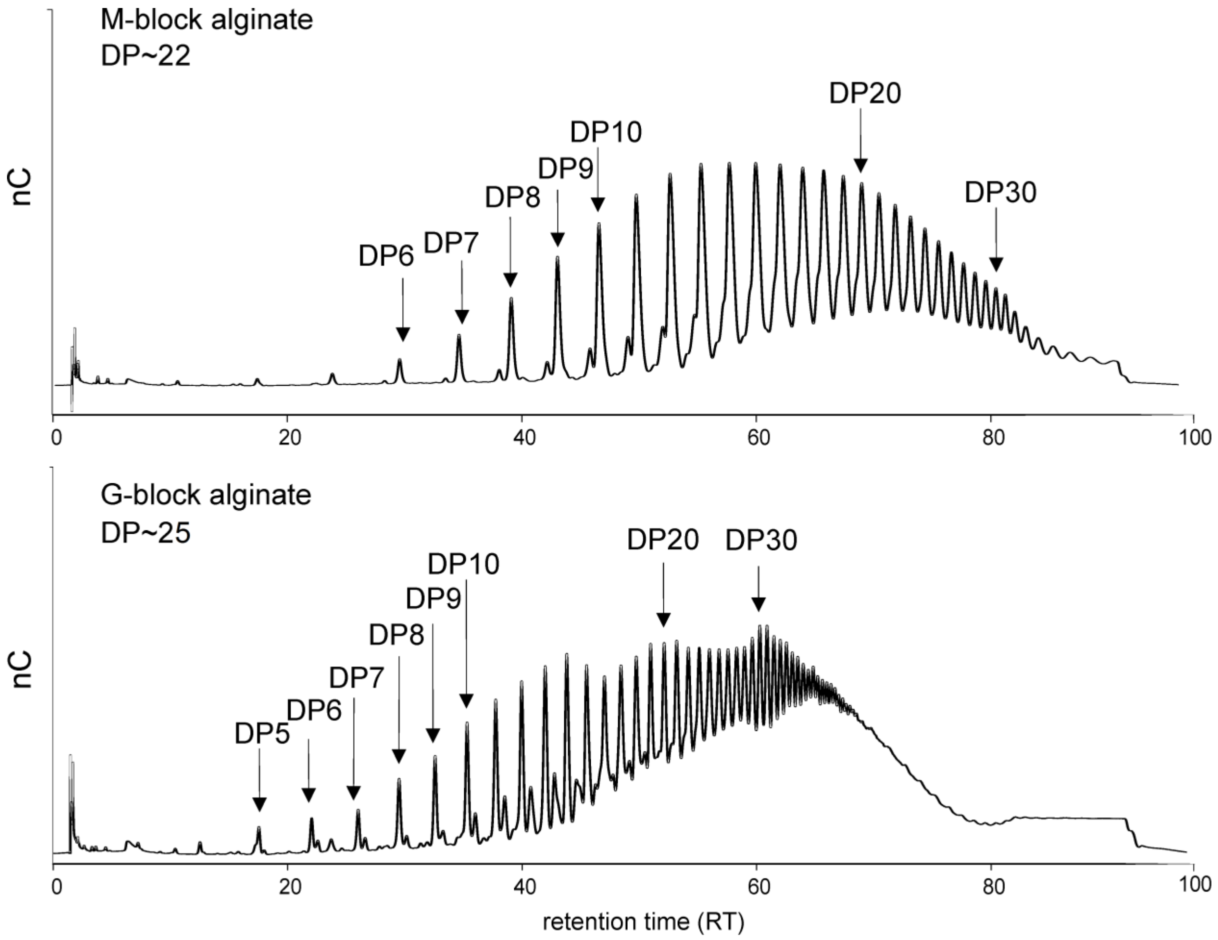

Supplementary Figure S6. HPAEC-PAD profiles of commercially available M-block alginate (mannuronan, DP~22) and G-block alginate (guluronan, DP~25).
