# Supplementary material for: Enzymatic depolymerization of alginate by two novel thermostable alginate lyases from Rhodothermus marinus
Source: Front Plant Sci. 2022 Sep 20;13:981602. doi: 10.3389/fpls.2022.981602 (PMC9530828; doi:10.3389/fpls.2022.981602)
Supplement: Supplementary file 9 [file Image_7.pdf]

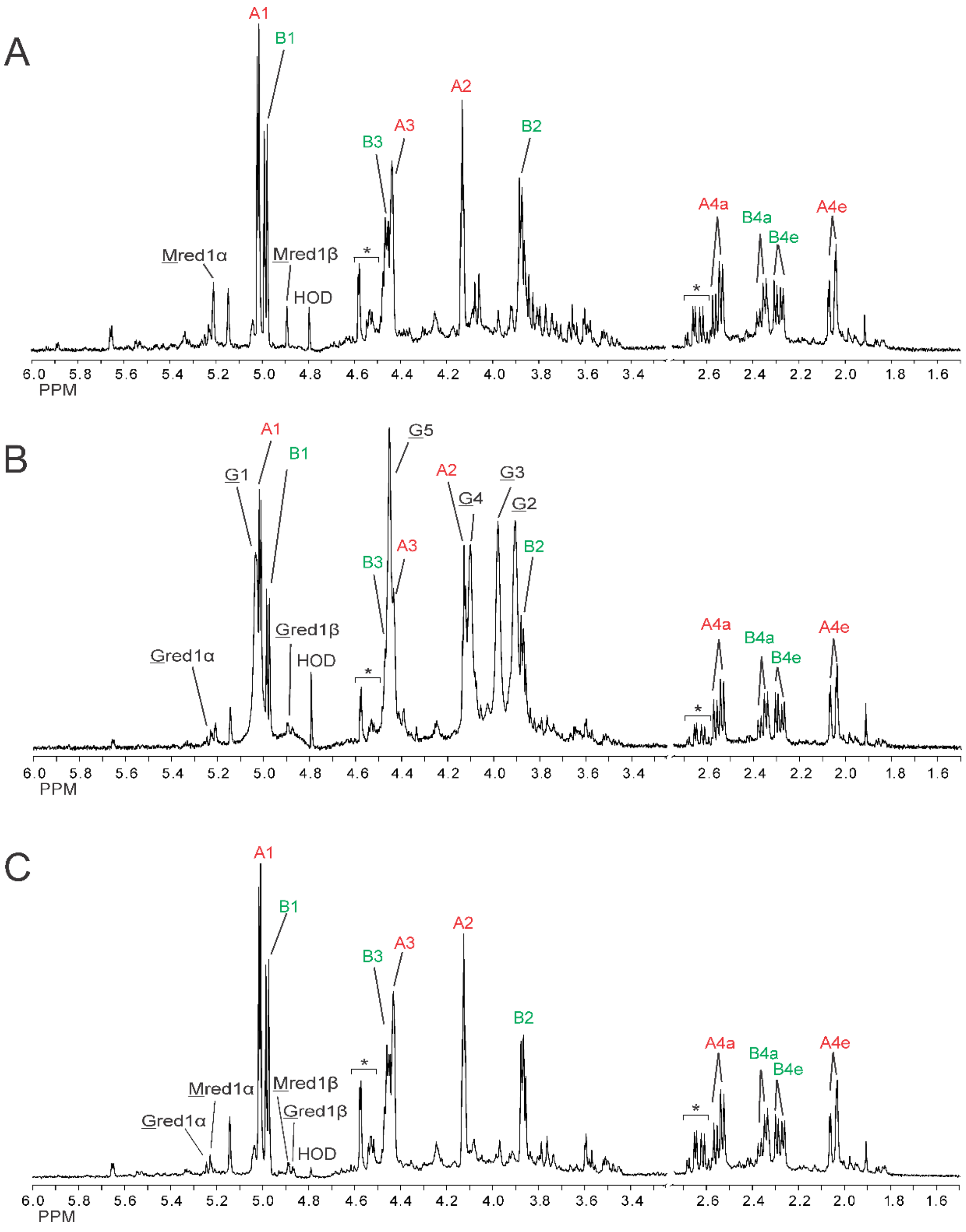


**Supplementary Figure S7.** 1D 500-MHz $^{1}H$ NMR spectra (300K, $D_2O$) of the saccharide mixtures of (A) the M-block alginate, (B) the G-block alginate, and (C) the low-viscosity *M. pyrifera* alginate, generated after 24 h incubations with the AlyRm4 enzyme. The A and B color-coding system (cyclic hemiacetal structures) is explained in Figure 8 of main text. (See also Supplementary Figure S8). * Traces of carboxylic acids.
