# Supplementary material for: Enzymatic depolymerization of alginate by two novel thermostable alginate lyases from Rhodothermus marinus
Source: Front Plant Sci. 2022 Sep 20;13:981602. doi: 10.3389/fpls.2022.981602 (PMC9530828; doi:10.3389/fpls.2022.981602)
Supplement: Supplementary file 10 [file Image_8.pdf]

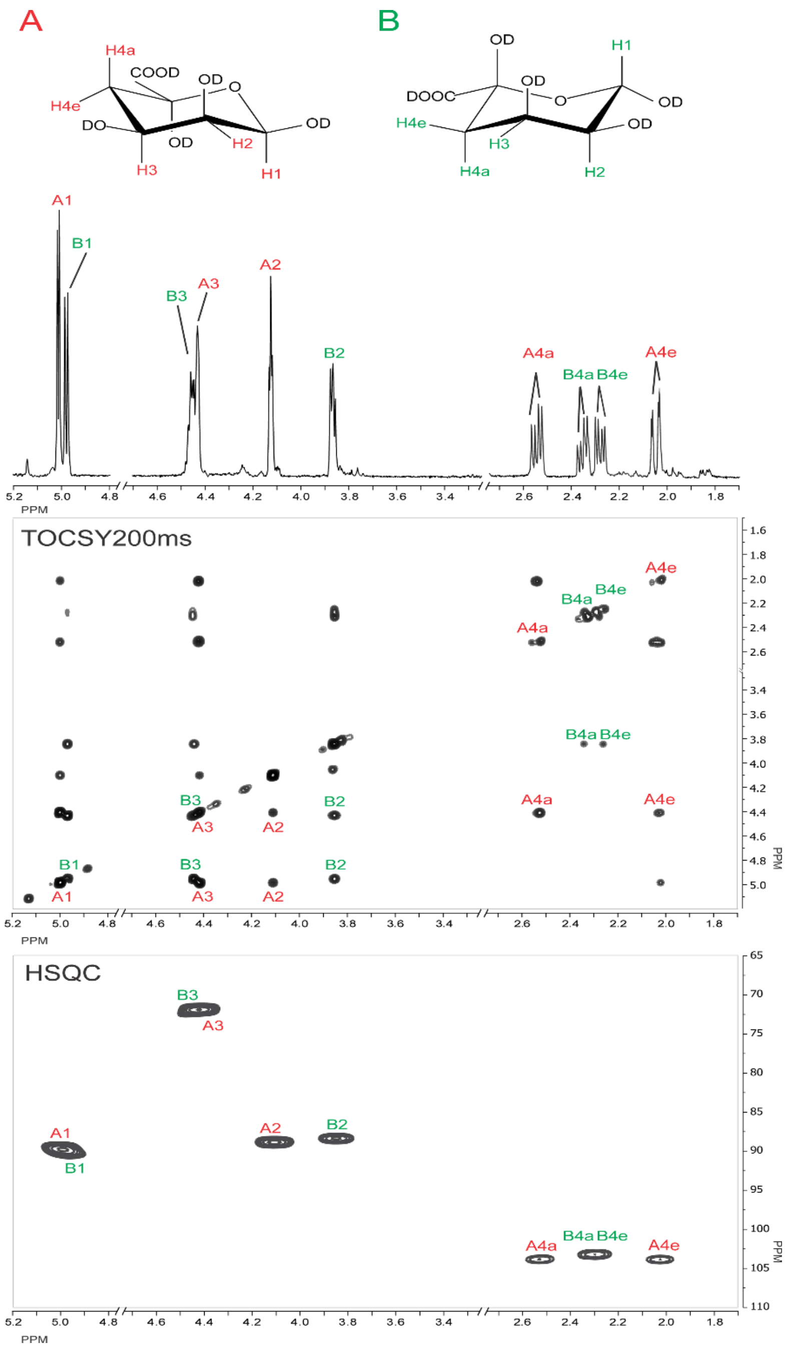

Supplementary Figure S8. 2D TOCSY and HSQC spectra of the cyclic stereoisomers A and B. (see also Figure 8 and Figure 9 in main text). Protons and carbons are assigned according to Enquist er al. (2014).
