# Supplementary material for: Enzymatic depolymerization of alginate by two novel thermostable alginate lyases from Rhodothermus marinus
Source: Front Plant Sci. 2022 Sep 20;13:981602. doi: 10.3389/fpls.2022.981602 (PMC9530828; doi:10.3389/fpls.2022.981602)
Supplement: Supplementary file 11 [file Image_9.pdf]

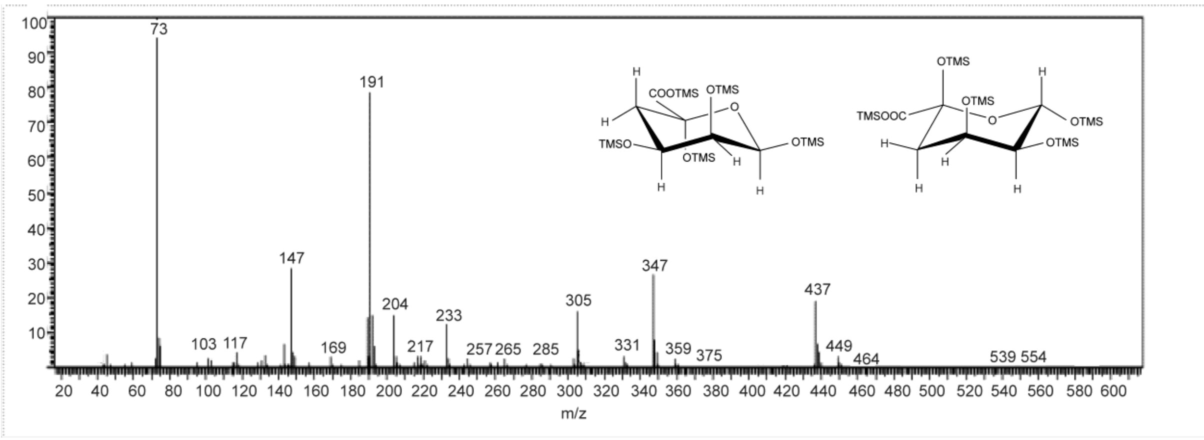

554	M	347	M – COOTMS – TMSOH
539	M – CH_3	305	TMSOCH=C(OTMS)CH=OTMS
464	M – TMSOH	217	TMSOCH=CHCHOTMS
449	M – CH_3 – TMSOH	204	TMSOCH=HCOTMS
437	M – COOTMS	191	TMSOCH=OTMS
359	M – CH_3 – 2x TMSOH	147	TMSOSi$(CH_3)_2$

Supplementary Figure S9. EI mass spectrum of trimethylsilylated (TMS) cyclic monomers A and B with fragment assignments. – means minus.
