# Supplementary material for: Enzymatic depolymerization of alginate by two novel thermostable alginate lyases from Rhodothermus marinus
Source: Front Plant Sci. 2022 Sep 20;13:981602. doi: 10.3389/fpls.2022.981602 (PMC9530828; doi:10.3389/fpls.2022.981602)
Supplement: Supplementary file 12 [file Image_10.pdf]

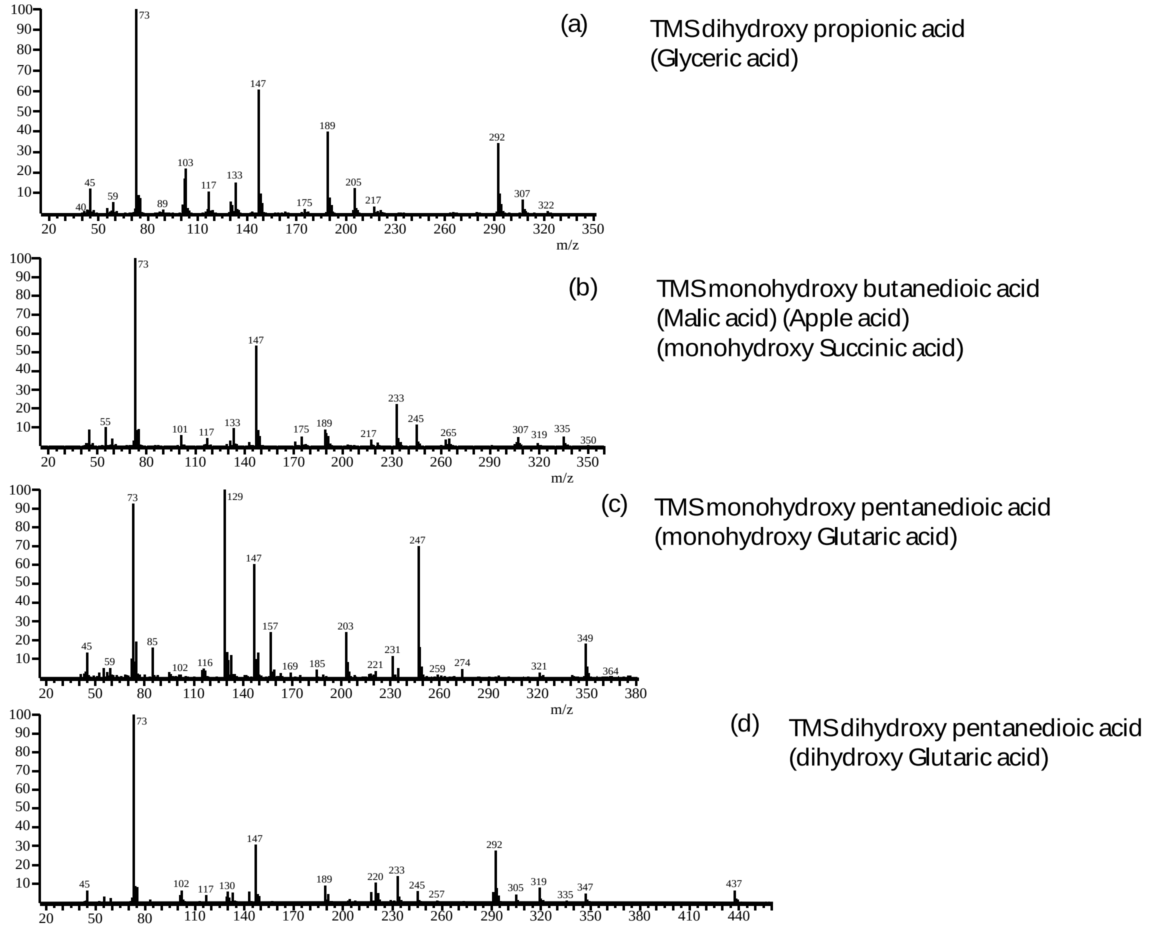

Supplementary Figure S10. EI-mass spectra of some TMS organic acids found in the product mixture of *M. pyrifera* alginate after incubation with AlyRm4 enzyme.
